# A Cascading Mean-Field Approach to the Calculation of Magnetization Fields in Magnetoactive Elastomers

**DOI:** 10.3390/polym13091372

**Published:** 2021-04-22

**Authors:** Dirk Romeis, Marina Saphiannikova

**Affiliations:** Leibniz-Institut für Polymerforschung Dresden e.V., Hohe Strasse 6, 01069 Dresden, Germany; grenzer@ipfdd.de

**Keywords:** magnetic polymers, magneto-active elastomers, dipole approximation, dipole model, self-consistent field, magnetization field

## Abstract

We consider magnetoactive elastomer samples based on the elastic matrix and magnetizable particle inclusions. The application of an external magnetic field to such composite samples causes the magnetization of particles, which start to interact with each other. This interaction is determined by the magnetization field, generated not only by the external magnetic field but also by the magnetic fields arising in the surroundings of interacting particles. Due to the scale invariance of magnetic interactions ($O(r^{-3})$ in d=3 dimensions), a comprehensive description of the local as well as of the global effects requires a knowledge about the magnetization fields within individual particles and in mesoscopic portions of the composite material. Accordingly, any precise calculation becomes technically infeasible for a specimen comprising billions of particles arranged within macroscopic sample boundaries. Here, we show a way out of this problem by presenting a greatly simplified, but accurate approximation approach for the computation of magnetization fields in the composite samples. Based on the dipole model to magnetic interactions, we introduce the cascading mean-field description of the magnetization field by separating it into three contributions on the micro-, meso-, and macroscale. It is revealed that the contributions are nested into each other, as in the Matryoshka’s toy. Such a description accompanied by an appropriate linearization scheme allows for an efficient and transparent analysis of magnetoactive elastomers under rather general conditions.

## 1. Introduction

Magnetoactive elastomers (MAEs), also often denoted as magneto-rheological or magneto-sensitive elastomers, represent a very promising class of field-controllable functional polymer materials. Their magneto-mechanical properties can undergo plenty of different changes and effects like magnetodeformation and anisotropic changes in dynamic and static mechanical moduli depending on strength, orientation, or modulation of the external magnetic field [1,2,3,4,5,6,7,8,9,10,11,12,13,14,15,16]. The specific constitution of an MAE sample itself can also be quite versatile, ranging from the usage of magnetically hard or magnetically soft filler particles to mixtures of both types [17,18,19]. In the following we consider the filler particles as magnetizable, i.e., they display no magnetization in the absence of a magnetic field, being produced from an ideal magnetically soft material. Additionally, different compositions of the polymeric material forming the elastic matrix can have substantial influence on the composite behavior under the applied field. Finally, it is well known that not only the bare amount of magnetic filler content, but also its arrangement into isotropic or anisotropic structures as well as the macroscopic form of the sample itself is of great importance for the control of the effective material behavior. Quite a variety of technical implementations and applications have been proposed and implemented so far. For example, MAEs can be used as a working part in actuators and sensors [20,21,22], energy harvesting devices [23,24,25], micro-robots [26] and -pumps [27], in prosthetic and orthotic devices [28], as well as in ophthalmologic magnetic fixators [29,30,31].

Although broadly speaking, one might consider MAEs just as magnetic rubber, the modeling and subsequent comprehension of MAEs is quite complex and challenging. According to our understanding, one of the essential reasons for this lies in the very nature of magnetic interactions. On distance *r* they scale like $O(r^{-3})$ in d=3 dimensions. Thus, the local arrangement of the nearest neighbor particles is equivalently as important as the macroscopic form of the entire sample [32]. This leads to an interesting interplay between particle microstructure and macroscopic shape effect, determining the effective deformation behavior in the applied magnetic field [33]. Yet, the theory presented in our previous works [32,33,34] so far has been limited to essential simplifications, such as the linear magnetization behavior, spheroidal sample forms, and/or simple lattice-like particle microstructures. We introduced such assumptions as they allowed to decouple the short- and long-range dipole-dipole interactions, compute their contribution separately, and finally recombine them into a unified approach. The crucial point within such an approach is that we obtain the average magnetization among all particles self-consistently, in dependence of the sample parameters, and derive subsequently the magnetic energy as a function of magnetodeformation and local particle rearrangements [32,33,34,35]. Minimizing the total free energy, it is possible then to predict the magneto-mechanical behavior of spheroidal samples in a wide range of situations [33,34]. For example, the magneto-induced stress is shown to increase with decreasing anisotropy of a spheroidal sample both for isotropic and columnar-like microstructures [33]. A similar effect has been confirmed recently for the cylindrically-shaped samples [36]. The deformational (and magnetizational) behavior of samples, containing stochastically isotropic and helical microstructures, is found to be in a remarkable agreement with the results of explicit micro-continuum mechanical modeling [35,37].

Unfortunately, in more realistic situations, i.e., beyond the linear magnetization approximation and for general sample shapes or particle microstructures, the short- and the long-range dipole interactions are inherently intertwined through complex functional dependencies. As a consequence for experimentally relevant conditions the magnetization field on a micro- and on mesoscale for an MAE can only be calculated accurately upon accounting for the macroscale simultaneously. Such a task is clearly beyond any computational resources, as it effectively requires a microscopic model of the entire macroscopic sample.

In the present work we develop an approximate solution scheme to this problem, examining the dipole-dipole interactions between magnetizable particles in more general situations. The scheme envolves a cascading mean-field description of the magnetization field followed by an appropriate linearization of the magnetization function. This allows to greatly decouple the effects coming into the magnetization field from the micro-, meso-, and macroscale in the composite samples with arbitrary microstructures and shapes. The paper is organized in the following way. First, we derive our approximation scheme in general form in Section 2. Here, we also analyze implications for linear and for saturating magnetization behavior and highlight possible interpretations of the results. To prove its appropriability we explicitly apply the scheme in Section 3. We consider two example situations for microstructure calculations and additionally determine the mesoscopic effects due to the macroscopic shape of a cylindrical sample. Finally, in Section 4 we formulate the conclusion.

## 2. The Cascading Mean Field Approach

Apparently, the driving force determining the behavior of an MAE sample under an external magnetic field has its origin in the overall changes of magnetic energy. This energy takes the following general form [33,38]:

(1) $$U_{mag}\;=\;\mu_{0}\;\;\int_{V_{S}}\;\;d^{3}r\left\{-\;\int_{0}^{H}\;\;M\;\cdot \;dH+\frac{1}{2}M\;\cdot \;\left(H-H_{0}\right)\right\}\;,$$

where $\mu_{0}$ is the vacuum permeability and $V_{S}$ is the volume of the sample. The magnetization field M(r) within a particle inclusion located at position r is assumed to be induced by the local magnetic field H(r), whereas the externally applied magnetic field is described by the constant vector $H_{0}$. To be able to calculate the magnetic energy, two basic inputs are necessary: (1) What kind of the particle microstructure is embedded inside the sample volume, and (2) which magnetization function describes the behavior of inclusions. As we mentioned in the introduction, an ideal magnetically soft behavior will be considered in the present work, following the most common experimental situation [30,39,40]. Having this basic information, the task of computing the magnetic energy stays highly nontrivial due to the presence of two unknown fields M and H in Equation (1), which should be calculated self-consistently. In particular, non-linear magnetization behavior, quite relevant in most experimental situations, makes the computation of magnetic energy and thus a prediction of magneto-mechanical properties technically infeasible. This will become clear from the considerations presented in the following session.

### 2.1. The Dipole Model

In the following let N be the total number of magnetizable particles distributed somehow within the macroscopic MAE sample. We assume a constant magnetization field within each of the inclusions, such that magnetic interactions among all the particles are described in terms of dipole fields. This assumption represents the so-called dipole model [33,41,42,43,44,45,46,47,48,49,50,51]. It is best suited for inclusions with the shape close to a spherical one, because a homogeneously magnetized sphere generates a dipole field in its exterior exactly. Neighboring inclusion particles should also remain at some distance from each other to assure a homogeneous field over the extent of each particle. This minimum center-to-center distance was found to be around $3r_{p}$, with $r_{p}$ being the particle radius [38]. This corresponds to a maximum of about 20–30% of the volume fraction of well-dispersed particles in an MAE sample [33]. However, for any arbitrarily shaped inclusions or for particles coming closer to each other the dipole approach can likewise be motivated as a first approximation. In the most general form the calculation of the local magnetization field within any inclusion a∈N of the sample follows from:

(2) $$M_{a}=L\left(H_{a}\right)\;.$$

Here, L(·) denotes the material magnetization function of the inclusions and will be specified in the following. For instance in case of isotropic linear magnetization, it simply reads $M_{a}=\chi H_{a}$, with magnetic susceptibility χ. In the framework of the dipole model, the local magnetic field $H_{a}$ is obtained via:

(3) $$H_{a}=H_{0}-{\hat{\nu }}_{a}\cdot M_{a}+\sum_{b\neq a}^{N}{\hat{g}}_{ab}\cdot M_{b}\;.$$

The externally applied magnetic field $H_{0}$ is assumed to be homogeneous over the extent of the MAE sample. For an arbitrarily-shaped magnetizable particle the tensor parameter ${\hat{\nu }}_{a}$ represents the self-demagnetization factor of inclusion *a*. In Equation (3) we defined the tensorial dipole operator between the particles *a* and *b* as:

(4) $${\hat{g}}_{ab}:=\frac{v_{b}}{4\pi }\frac{3r_{ab}r_{ab}-r_{ab}^{2}\hat{I}}{r_{ab}^{5}}\;.$$

The vector $r_{ab}=r_{b}-r_{a}$ describes the distance vector a↔b, $\hat{I}$ denotes the second order unit tensor, and $v_{b}$ accounts for the volume of inclusion *b*.

The fact that the sum in Equation (3) runs over all inclusions in the entire sample is due to the long-range nature of the magnetic interactions, here approximated in terms of the dipole field, Equation (4). Accordingly, even within the (simplifying) dipole approximation the calculation of the magnetic energy in an MAE sample requires a self-consistent solution of a macroscopic number (here 3N in 3 dimensions) of coupled equations, Equations (2) and (3) ∀a∈N. We note, that already in the elementary case of linear magnetization (M=χH), and a respective set of linear equations, a straightforward solution for any macroscopic sample ($N∼O(10^{9\ldots 12})$) is technically not feasible as it requires the calculation, storage, and inversion of a (3N×3N)-Matrix.

### 2.2. Introducing the Cascading Mean Field Approach (*‘Matryoshka’ Scheme*)

**Figure 1:**
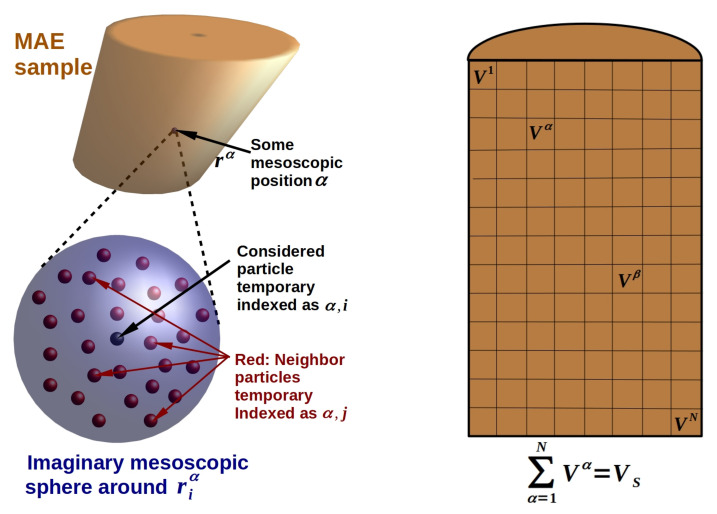
(**Left**) Sketch of the decomposition of an MAE sample into short- and long-range effects. (**Right**) Formal discretization of sample volume $V_{S}$ into mesoscopic portions $V^{\alpha }$, α∈[1,N]. On such scales any particle microstructure appears a homogeneous continuous distribution.

For specially selected and simplified situations the problem of the equivalence of short- and long-range magnetic interactions has been solved in various previous publications [32,33,34,36,42,52]. The main strategy is based on the understanding that beyond a certain distance from any inclusion the precise local particle arrangement is no more resolvable. It appears as a homogeneously smeared continuous distribution at larger distances. Usually this ‘critical’ resolution range is around 10 times the average nearest neighbor distance [32]. This behavior simply resembles the fact that from a sufficiently far distance any microstructure appears homogeneous [32,33,34,35]. The idea of decoupling the micro- and macro-effect has been outlined previously [32,33] and it is sketched on the left side in Figure 1. We emphasize that in the macroscopic limit such a decomposition is inherently exact.

In a first step we divide the macroscopic MAE sample into *N* mesoscopic partial volumes $V^{\alpha },\;\alpha =1,N$. The length scale of such mesoscopic portions is defined as very small compared to the macroscopic sample size, but very large compared to typical distances among neighboring particles, i.e., the local microstructure. The macroscopic limit assures, so to say, that on a mesoscopic scale any sample volume $V_{S}$ may be perfectly resolved upon increasing *N* to an arbitrary large number, whereas any particle microstructure appears completely diffuse. On the right in Figure 1 a formal portioning of an MAE sample is sketched. We emphasize that each mesoscopic volume $V^{\alpha }$ appears from any other volume $V^{\beta }$ as a homogeneous material point containing the locally averaged volume fraction $\phi^{\alpha }$ of magnetizable material.

In the following we denote the limiting range around any reference particle where the microstructure starts to appear homogeneous as the mesoscopic sphere. One prominent consequence of decoupling the micro- and macro-effect is the capability to provide even analytically representable expressions for the magnetic energy of an entire macroscopic MAE sample. It can be shown how the macroscopic effect, following from the given form of the MAE sample, and the microscopic effect, following from the local particle structure, are intertwined and how this interplay affects the magneto-mechanical properties of MAEs [32,33]. Nevertheless, although the virtual implementation of the mesoscopic sphere is generally valid, in previous works we introduced additional simplifying assumptions in order to obtain a (partially) analytical tractable form. In particular, we want to mention that we were able to obtain self-consistently the locally varying magnetization field, arising due to the particle microstructure, and this was done independent of the actual macroscopic aspect ratio of the sample. Such a decomposed representation of the problem can only be achieved for the special case of a linear magnetization scheme and assuming an ellipsoidal sample form. Whenever one considers a more general, i.e., non-linear, magnetization function in Equation (2) a decomposition is no longer achievable.

In the present work we want to further generalize our existing approach towards less restrictive assumptions and thereby also towards more realistic situations as likely relevant for practical purposes. Adapting the idea of a mesoscopic sphere to the general case, we may rewrite the formal relation in Equation (3) as:

(5) $$H_{i}^{\alpha }=H_{0}-{\hat{\nu }}_{i}^{\alpha }\cdot M_{i}^{\alpha }+\sum_{\beta \neq \alpha }^{N}\phi^{\beta }\;{\hat{g}}^{\alpha \beta }\;\cdot \;M^{\beta }+\sum_{j\neq i}^{n^{\alpha }}{\hat{g}}_{ij}^{\alpha }\;\cdot \;M_{j}^{\alpha }\;.$$

The notation introduced in Equation (5) strictly separates micro-structural components, here with lower indices (i,j), from mesoscopic form components, here upper indices (α,β). Accordingly, parameter *N* limiting the first sum in Equation (5) corresponds to the number of mesoscopic volume elements (see Figure 1) to which the macroscopic sample has been formally discretized. Since $\sum_{\alpha =1}^{N}V^{\alpha }=V_{S}$ the total, or macroscopically averaged, volume fraction of magnetizable inclusions in the sample, ϕ, amounts to:

(6) $$\phi =\frac{1}{V_{S}}\sum_{\alpha =1}^{N}\phi^{\alpha }V^{\alpha }\;.$$

Furthermore, the field quantities follow a hierarchical order, where ${\left(\cdot \right)}_{i}^{\alpha }$ denotes the corresponding field in the particle located mesoscopically at position α within the macroscopic sample and which is microscopically identified as the i−th particle within the local microstructure, see Figure 1. Regularly, in the macroscopic (or thermodynamic) limit the index α is a continuous variable scanning the macroscopic form of the sample. Thus, the sum $\sum_{\beta =1}^{N}$ represents effectively an integral over the sample volume $V_{S}$. For convenience we keep here its discrete form and note that an explicit numerical evaluation of arbitrarily shaped samples requires a corresponding volume discretization anyways. The tensors $\hat{g}$ in Equation (5) are adapted accordingly:

(7) $${\hat{g}}^{\alpha \beta }:=\frac{V^{\beta }}{4\pi }\frac{3r^{\alpha \beta }r^{\alpha \beta }-{r^{\alpha \beta }}^{2}\hat{I}}{{r^{\alpha \beta }}^{5}}\;,$$

(8) $${\hat{g}}_{ij}^{\alpha }:=\frac{v_{j}^{\alpha }}{4\pi }\frac{3r_{ij}^{\alpha }r_{ij}^{\alpha }-{r_{ij}^{\alpha }}^{2}\hat{I}}{{r_{ij}^{\alpha }}^{5}}\;.$$

In Equation (5) we present the relation in a very general form. So we allow each inclusion to be of a different size, $v_{i}^{\alpha }$, and to have an individual self-demagnetization factor, ${\hat{\nu }}_{i}^{\alpha }$. The average local particle density may vary, $\phi^{\alpha }$, and therefore the number of particles representing an actual local microstructure, $n^{\alpha }$, as well as the micro-structural dipole operator, ${\hat{g}}_{ij}^{\alpha }$, may be different. In principle, one could here even allow for a locally varying external magnetic field, $H_{0}\to H_{0}^{\alpha }$.

The present notation greatly generalizes our previous studies. Nevertheless, we introduce several appropriate simplifications since we do not account for all possible situations here. In the following the external magnetic field is strictly homogeneous. The particles immersed in the MAE are identical spheres, they are monodisperse in size with $v_{i}^{\alpha }=v$ and ${\hat{\nu }}_{i}^{\alpha }=\hat{\nu }=\frac{1}{3}\hat{I};$ ∀i,α. Furthermore, we consider a homogeneous particle density on a mesoscopic scale, $\phi^{\alpha }=\phi$ and $n^{\alpha }=n;$ ∀α. Finally, for simplicity and to keep the derivation possibly short, we constrain to ${\hat{g}}_{ij}^{\alpha }={\hat{g}}_{ij};\forall \alpha$. Thus, we consider only one single type of microstructure present in the entire volume of an actual sample. With the aforementioned simplifications we finally come to the following relation:

(9) $$H_{i}^{\alpha }=H_{0}-\hat{\nu }\cdot M_{i}^{\alpha }+\phi \sum_{\beta \neq \alpha }^{N}{\hat{g}}^{\alpha \beta }\;\cdot M^{\beta }+\sum_{j\neq i}^{n}{\hat{g}}_{ij}\cdot M_{j}^{\alpha }\;.$$

Actually, the cases of varying mesoscopic densities $\phi^{\alpha }$ and/or of varying microstructures ${\hat{g}}_{ij}^{\alpha }$ in different sections α of the sample appear practically relevant and also quite interesting for an even more comprehensive theoretical description of MAE in external magnetic fields. Perhaps this will be for future consideration. Nevertheless, for a freshly synthesized MAE sample it is reasonable to assume that the particles are similarly distributed all over its volume and thus $\phi^{\alpha }=\phi$ and ${\hat{g}}_{ij}^{\alpha }\approx {\hat{g}}_{ij}\;;\forall \alpha$. Upon applying an external magnetic field, the local field deforming the specimen may vary on macroscopic scales resulting in deflection, buckling, or any kind of asymmetric macroscopic distortion. Accordingly, the microstructure may rearrange differently in the different parts of the sample. In the present work we neglect such effects and assume uniform rearrangements on a macroscopic scale. We also want to note that in the course of applying an external magnetic field the variation of a particle microstructure within an individual sample has been often disregarded in literature [40,53,54].

**Figure 2:**
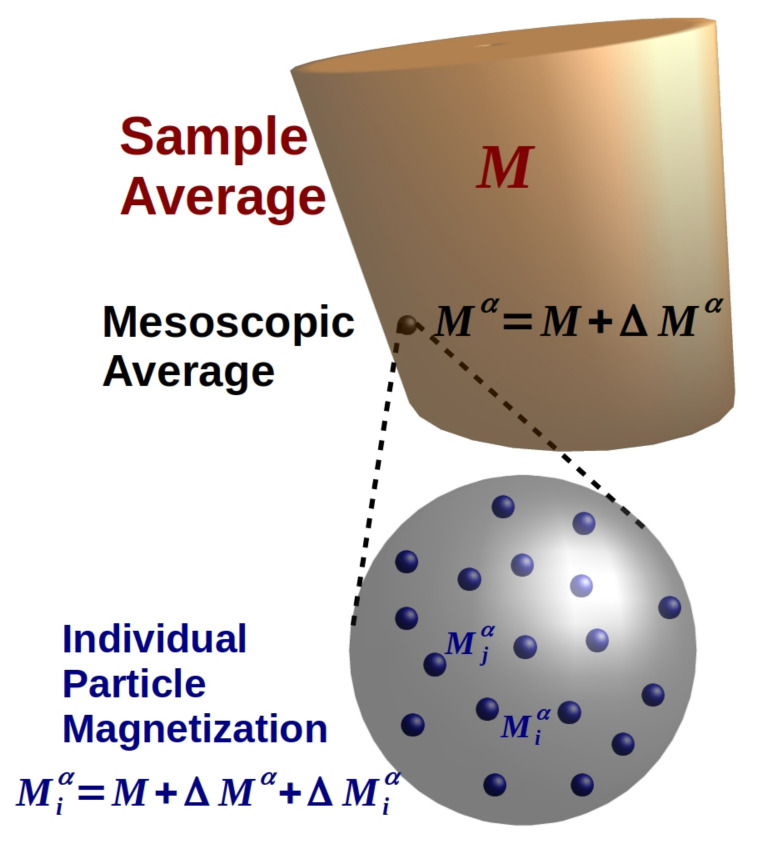
Splitting the ‘actual’ individual particle magnetization $M_{i}^{\alpha }$ into the average magnetization among all particles M (macroscopic) plus deviations $\Delta M^{\alpha }$ (mesoscopic) and $\Delta M_{i}^{\alpha }$ (microscopic).

Still, Equation (9) for a local magnetic field should be calculated self-consistently when inserted in Equation (2). We make use of what we call a Cascading Mean-Field description and implement the following notation:

(10) $$M_{i}^{\alpha }=M+\Delta M^{\alpha }+\Delta M_{i}^{\alpha }\;,$$

where

(11) $$M:=\frac{1}{Nn}\sum_{\alpha }^{N}\left(\sum_{i}^{n}M_{i}^{\alpha }\right)=\frac{1}{N}\sum_{\alpha }^{N}M^{\alpha }\;$$

represents the macroscopic magnetization averaged over all particles in the sample and

(12) $$M^{\alpha }:=M+\Delta M^{\alpha }=\frac{1}{n}\sum_{i}^{n}M_{i}^{\alpha }\;,$$

denotes the mesoscopic magnetization averaged over the particles found in the mesoscopic volume element $V^{\alpha }$. Note, that the deviations $\Delta M^{\alpha }$ and $\Delta M_{i}^{\alpha }$ are introduced such that $\sum_{\alpha }^{N}\Delta M^{\alpha }=0$ and $\sum_{i}^{n}\Delta M_{i}^{\alpha }=0$. The cascading scheme is sketched in Figure 2. It visualizes the three contributions on the micro-, meso-, and macroscale and how the contributions to the magnetization field are nested into each other, similarly to how it is performed in the Matryoshka’s toy.

Additionally, we introduce new tensorial operators:

(13) $${\hat{G}}_{i}:=\sum_{j\neq i}^{n}{\hat{g}}_{ij}$$

and

(14) $${\hat{G}}^{\alpha }:=\sum_{\beta \neq \alpha }^{N}{\hat{g}}^{\alpha \beta }.$$

We define the average microscopic structure factor ${\hat{G}}_{\mathrm{micro}}$ and the average macroscopic sample form factor ${\hat{G}}_{\mathrm{macro}}$ following our notation via:

(15) $${\hat{G}}_{\mathrm{micro}}:=\frac{1}{n}\sum_{i}^{n}{\hat{G}}_{i}\;,$$

and

(16) $${\hat{G}}_{\mathrm{macro}}:=\frac{1}{N}\sum_{\alpha }^{N}{\hat{G}}^{\alpha }\;.$$

That way we may formalize the $\hat{G}$s analogous to Equation (10) as:

(17) $${\hat{G}}_{i}={\hat{G}}_{\mathrm{micro}}+\Delta {\hat{G}}_{i}$$

and

(18) $${\hat{G}}^{\alpha }={\hat{G}}_{\mathrm{macro}}+\Delta {\hat{G}}^{\alpha }.$$

In addition, from Equation (18), resp. Equation (17), follows that the corresponding sum over α of $\Delta {\hat{G}}^{\alpha }$, resp. over *i* of $\Delta {\hat{G}}_{i}$, is equal to zero.

Using the notation convention, given by Equations (10)–(17), we rewrite Equation (9):

(19) $$\begin{array}{c} H_{i}^{\alpha }=H_{0}+\left(\phi {\hat{G}}_{\mathrm{macro}}+{\hat{G}}_{\mathrm{micro}}-\hat{\nu }\right)\;\cdot \;M+\left(\phi \Delta {\hat{G}}^{\alpha }\;+\Delta {\hat{G}}_{i}\right)\cdot M\\ +\left({\hat{G}}_{\mathrm{micro}}+\Delta {\hat{G}}_{i}-\hat{\nu }\right)\cdot \Delta M^{\alpha }+\phi \sum_{\beta \neq \alpha }^{N}{\hat{g}}^{\alpha \beta }\cdot \Delta M^{\beta }-\hat{\nu }\cdot \Delta M_{i}^{\alpha }+\sum_{j\neq i}^{n}{\hat{g}}_{ij}\;\cdot \;\Delta M_{j}^{\alpha }\;. \end{array}$$

The mean value of Equation (19) over all particles gives the average magnetic field H. Noting that the tensorial dipole operators are symmetric with respect to their indices, i.e., ${\hat{g}}^{\alpha \beta }={\hat{g}}^{\beta \alpha }$ and ${\hat{g}}_{ij}={\hat{g}}_{ji}$, the sum $\frac{1}{Nn}\sum_{\alpha }^{N}\sum_{i}^{n}$ of Equation (19) reads:

(20) $$H=H_{0}+\left(\phi {\hat{G}}_{\mathrm{macro}}+{\hat{G}}_{\mathrm{micro}}-\hat{\nu }\right)\;\cdot \;M+\phi \sum_{\alpha }^{N}\frac{\Delta {\hat{G}}^{\alpha }\;\cdot \;\Delta M^{\alpha }}{N}+\sum_{i}^{n}\frac{\Delta {\hat{G}}_{i}\;\cdot \;{\langle \Delta M_{i}\rangle }_{S}}{n}\;.$$

Here, ${\langle \Delta M_{i}\rangle }_{S}$ denotes the mean deviation of the individual particle magnetization, $\Delta M_{i}^{\alpha }$, from the mesoscopic magnetization, $M^{\alpha }$, as averaged over the sample volume:

(21) $${\langle \Delta M_{i}\rangle }_{S}:=\frac{1}{N}\sum_{\alpha }^{N}\Delta M_{i}^{\alpha }\;.$$

At this point we shortly want to discuss the role of the magnetization function L(·) in Equation (2). Presuming an isotropic linear magnetization behavior would allow us to keep following a mathematically exact derivation. The reason is that then the average magnetization M and the average magnetic field H are related linearly allowing a direct insertion of Equation (20) in the magnetization function, M=χH. Subsequently, it would be possible to decouple to a great extent the (still exact) relations on the different length scales, i.e., achieving a partition into two almost independent sets of self-consistent equations: One for the macroscopic sample form and one for the microscopic particle structure. The case of isotropic linear magnetization will be discussed in detail in Section 2.4.

### 2.3. General Magnetization and Linearization

To proceed from Equation (20), the central difficulty is due to the inequality M≠L(H) for a general, i.e., non-linear, magnetization function. In order to profit from the implications of a linear relation we deploy a classical Taylor expansion, which we restrict to first order to yield a subsequent linearization of the magnetization function. This expansion shall be developed around an approximate average magnetic field A, which we define as follows:

(22) $$A:=H_{0}+\left(\phi {\hat{G}}_{\mathrm{macro}}+{\hat{G}}_{\mathrm{micro}}-\hat{\nu }\right)\;\cdot \;M\;.$$

The approximate A only accounts for the first two terms of the exact H in Equation (20). We motivate the neglect of the last two terms in Equation (20) by the following reason. One immediately notes that these terms represent the microscopic, resp. mesoscopic, corrections to the macroscopic sample averages and involve the solution/knowledge of a huge system of equations, i.e., since they are in fact coupled: O((3N×3N)(3n×3n)). In contrast, the approximate solution for M, resp. A, in Equation (22) only involves 3 equations, one for each spatial direction of the global M. From the computational point of view this represents an enormous benefit. To neglect any correction due to alterations $\Delta \hat{G}$ and/or ΔM represents the leading order approximation in our cascading mean-field approach.

Beyond the leading order we apply a Taylor expansion around A and approximate the magnetization $M_{i}^{\alpha }=L\left(H_{i}^{\alpha }\right)$ in the particle *i* of mesoscopic domain α as:

(23) $$\begin{array}{c} M_{i}^{\alpha }\approx L\left(A\right)+\nabla_{A}L\left(A\right)\cdot \{\left(\phi \Delta {\hat{G}}^{\alpha }\;+\Delta {\hat{G}}_{i}\right)\cdot M+\left({\hat{G}}_{\mathrm{micro}}+\Delta {\hat{G}}_{i}-\hat{\nu }\right)\cdot \Delta M^{\alpha }\\ -\hat{\nu }\cdot \Delta M_{i}^{\alpha }+\phi \sum_{\beta \neq \alpha }^{N}{\hat{g}}^{\alpha \beta }\cdot \Delta M^{\beta }+\sum_{j\neq i}^{n}{\hat{g}}_{ij}\;\cdot \;\Delta M_{j}^{\alpha }\}\;. \end{array}$$

Taking on both sides in Equation (23) the mesoscopic average over the microstructure, $\frac{1}{n}\sum_{i}^{n}$, we get:

(24) $$\begin{array}{c} M^{\alpha }\approx L\left(A\right)+\nabla_{A}L\left(A\right)\cdot \{\phi \Delta {\hat{G}}^{\alpha }\cdot M+\left({\hat{G}}_{\mathrm{micro}}-\hat{\nu }\right)\cdot \Delta M^{\alpha }\\ +\phi \sum_{\beta \neq \alpha }^{N}{\hat{g}}^{\alpha \beta }\cdot \Delta M^{\beta }+\sum_{j}^{n}\frac{\Delta {\hat{G}}_{j}\cdot \Delta M_{j}^{\alpha }}{n}\}\;. \end{array}$$

Finally, the mean magnetization field in the entire sample is obtained upon taking on both sides in Equation (24) the macroscopic average over the sample form, $\frac{1}{N}\sum_{\alpha }^{N}$. This yields:

(25) $$M\approx L\left(A\right)+\nabla_{A}L\left(A\right)\cdot \left\{\phi \sum_{\beta }^{N}\frac{\Delta {\hat{G}}^{\beta }\cdot \Delta M^{\beta }}{N}+\sum_{j}^{n}\frac{\Delta {\hat{G}}_{j}\cdot {\langle \Delta M_{j}\rangle }_{S}}{n}\right\}\;.$$

Here, we make use of the definition ${\langle \Delta M_{j}\rangle }_{S}$ introduced in Equation (21).

The equations above represent the linearized approximation for a general magnetization function L(·). Note, that in case of an isotropic linear magnetization, M=χH, as well as for anisotropic linear behavior, $M=\hat{\chi }\cdot H$, the relations are exact.

#### 2.3.1. The Local Magnetization within an Individual Particle

Subtracting both sides of Equation (24) from the corresponding sides of Equation (23), we end up with the following relation for the $\Delta M_{i}^{\alpha }$:

(26) $$\Delta M_{i}^{\alpha }\approx \nabla_{A}L\left(A\right)\cdot \left\{\Delta {\hat{G}}_{i}\cdot M^{\alpha }-\hat{\nu }\;\cdot \;\Delta M_{i}^{\alpha }+\sum_{j}^{n}\left(\;\;\left(1-\delta_{ij}\right){\hat{g}}_{ij}-\frac{\Delta {\hat{G}}_{j}}{n}\right)\;\cdot \;\Delta M_{j}^{\alpha }\right\}\;.$$

The form of Equation (26) immediately implies that within the present linearized approach $\Delta M_{i}^{\alpha }$ is proportional to $M^{\alpha }$ for all *i*. This fact greatly reduces the computational complexity of the problem. Note that the general solution for $\Delta M_{i}^{\alpha }$ can be obtained fully independent of the actual mesoscopic position α within a given sample form. We adopt a more compact notation by defining:

(27) $${\hat{\kappa }}_{ij}:=\left(1-\delta_{ij}\right){\hat{g}}_{ij}-\frac{\Delta {\hat{G}}_{j}}{n}\;.$$

The tensors ${\hat{\kappa }}_{ij}$ are exclusively determined by the micro-structural arrangement of the particles, i.e., by ${\hat{g}}_{ij}$. Furthermore, following the common notation for linear magnetization scheme, we may construct the prefactor in the r.h.s. of Equation (26) as a generalized susceptibility tensor:

(28) $${\hat{\chi }}_{A}:=\nabla_{A}L\left(A\right)\;,$$

and introduce thereby also an effective generalized susceptibility via:

(29) $${\hat{\chi }}_{A_{\mathrm{eff}}}:={\left(\hat{I}+{\hat{\chi }}_{A}\cdot \hat{\nu }\right)}^{-1}\cdot {\hat{\chi }}_{A}\;.$$

Accordingly, we rewrite Equation (26):

(30) $$\Delta M_{i}^{\alpha }\approx {\hat{\chi }}_{A_{\mathrm{eff}}}\cdot \left(\Delta {\hat{G}}_{i}\cdot M^{\alpha }+\sum_{j}^{n}{\hat{\kappa }}_{ij}\cdot \Delta M_{j}^{\alpha }\right).$$

Since each $\Delta M_{i}^{\alpha }$ consists of 3 components, Equation (30) represents a system of 3n linear equations. Traditionally, the solution of such a system may by obtained via a direct procedure, e.g., inverting the problem via Gaussian elimination. Let us introduce some generalized n×n matrices via:

(31a) $$\begin{array}{cc} {\left[c\right]}_{ij} & :={\hat{\chi }}_{A_{\mathrm{eff}}}\cdot {\hat{\kappa }}_{ij} \end{array}$$

(31b) $$\begin{array}{cc} {\left[I\right]}_{ij} & :=\hat{I}\delta_{ij} \end{array}$$

(31c) $$\begin{array}{cc} s & :={\left(I-c\right)}^{-1}\;. \end{array}$$

Note, that the entries of the generalized matrices are second-order tensors and correspondingly they effectively represent 3n×3n matrices. Then, the solution may be expressed formally as:

(32) $$\Delta M_{i}^{\alpha }\approx \left\{\sum_{j}^{n}\left(s_{ij}\cdot {\hat{\chi }}_{A_{\mathrm{eff}}}\cdot \Delta {\hat{G}}_{j}\right)\right\}\cdot M^{\alpha }.$$

Alternatively, the form of Equation (30) also allows for an iterative solution procedure starting with $\Delta M_{i}^{\alpha }\approx {\hat{\chi }}_{A_{\mathrm{eff}}}\cdot \Delta {\hat{G}}_{i}\cdot M^{\alpha }$ as the first estimate. In terms of the formalization in Equation (31) this yields:

(33) $$s=I+\left\{\sum_{k=1}^{\infty }{\left(c\right)}^{k}\right\}\;.$$

Note, the series in Equation (33) may be obtained upon expanding Equation (31c) in a Taylor series around $I={\left(I\right)}^{-1}$. The iteration scheme is only meaningful if the increasing powers ${\left(c\right)}^{k}$ rapidly diminish and, correspondingly, the series in Equation (33) converges. Indeed, for any distribution of particles this is true for two reasons. Firstly, since particles do not overlap we have $r_{ij}\geq d_{p}$, where $d_{p}$ denotes the particle diameter with $v=\pi d_{p}^{3}/6$, in Equation (8) and thus the entries of ${\hat{g}}_{ij}$, with i≠j, as well as those of $\Delta {\hat{G}}_{j}/n$, in Equation (27) are correspondingly small. Secondly, it is important to emphasize that it is the self-demagnetization term $\propto -\hat{\nu }$ which represents by far the major contribution in the right-hand side of Equation (26). Hence, upon introducing the generalized effective susceptibility ${\hat{\chi }}_{A_{\mathrm{eff}}}$ in Equation (29) and passing over to Equation (30) this self-demagnetization is implicitly, and exactly, taken into account. Furthermore, by definition, see Equation (29), the components of ${\hat{\chi }}_{A_{\mathrm{eff}}}$ are bound to moderate values even though the components of the generalized susceptibility itself, ${\hat{\chi }}_{A}=\nabla_{A}L\left(A\right)$, may become very large. In contrast to Equation (26), it is now ${\hat{\chi }}_{A_{\mathrm{eff}}}$ which is forming the prefactor in Equation (30), resp. in Equation (31a).

It is important to note that the computation of the generalized matrix s, either via some direct method or iteratively, using Equation (33), is completely independent of the mesoscopic position α within the sample. Nevertheless, we also note a fundamental drawback for magnetization functions L(·) beyond the ‘true’ linear magnetization scheme. Only in this case where M=χH, or general $M=\hat{\chi }\cdot H$, the here defined ${\hat{\chi }}_{A}$, Equation (28), and accordingly also the effective ${\hat{\chi }}_{A_{\mathrm{eff}}}$, Equation (29), are pure material parameters. They simply become ${\hat{\chi }}_{A}=\hat{\chi }$, resp. ${\hat{\chi }}_{A_{\mathrm{eff}}}={\hat{\chi }}_{\mathrm{eff}}$. Beyond linear magnetization functions these parameters depend on the approximate average magnetic field A, given by Equation (22). Accordingly, the matrix c, and consequently s in Equation (31) depend on A. Thus, whenever for example the particle content ϕ is altered, or the average macroscopic sample form factor ${\hat{G}}_{\mathrm{macro}}$ changes (likewise if ${\hat{G}}_{\mathrm{micro}}$ changes), or, most prominently, whenever just a different external magnetic field $H_{0}$ is applied, the computation, for example in Equation (32), must be repeated. This is not surprising, since within such a highly coupled system as an MAE sample in the applied field, the externally applied field becomes effectively a ‘material’ or internal parameter itself. To cut the matter short, each $H_{0}$ provokes a different material behavior and beyond the linear regime its role can not be decoupled, or resolved, from the other system parameters.

As a reminder, the leading order approximation is defined upon reducing Equations (20)–(22). In a first step beyond this leading order we will neglect the series in the brackets in Equation (33) and assume s≈I. With this approximation we may offer up some accuracy but at the same time we yet gain great generality of the approach, where most parameters are effectively decoupled allowing an efficient and transparent analysis of a very wide range of conditions and situations.

#### 2.3.2. The Mesoscopic Magnetization

Let us proceed with an equivalent derivation of the mesoscopic $\Delta M^{\alpha }$. Subtracting both sides of Equation (25) from the corresponding sides of Equation (24) we find:

(34) $$\begin{array}{c} \Delta M^{\alpha }\approx {\hat{\chi }}_{A}\;\cdot \;\{\phi \Delta {\hat{G}}^{\alpha }\cdot M+\left({\hat{G}}_{\mathrm{micro}}-\hat{\nu }\right)\;\cdot \;\Delta M^{\alpha }+\phi \sum_{\beta }^{N}\left(\;\;\left(1-\delta^{\alpha \beta }\right){\hat{g}}^{\alpha \beta }-\frac{\Delta {\hat{G}}^{\beta }}{N}\right)\;\cdot \;\Delta M^{\beta }\\ +\sum_{j}^{n}\frac{\Delta {\hat{G}}_{j}\cdot \left(\Delta M_{j}^{\alpha }-{\langle \Delta M_{j}\rangle }_{S}\right)}{n}\}\;. \end{array}$$

From Equation (32), we know that $\Delta M_{j}^{\alpha }\propto M^{\alpha }=M+\Delta M^{\alpha }$ and accordingly ${\langle \Delta M_{j}\rangle }_{S}\propto M$, with identical prefactors. Hence, the last line in Equation (34) transforms to:

(35) $$\sum_{j}^{n}\frac{\Delta {\hat{G}}_{j}\cdot \left(\Delta M_{j}^{\alpha }-{\langle \Delta M_{j}\rangle }_{S}\right)}{n}=\Delta {\hat{G}}_{\mathrm{micro}}\cdot \Delta M^{\alpha }\;,$$

where the tensor $\Delta {\hat{G}}_{\mathrm{micro}}$ is defined as:

(36) $$\Delta {\hat{G}}_{\mathrm{micro}}:=\frac{1}{n}\sum_{i,j}^{n}\left\{\Delta {\hat{G}}_{i}\cdot s_{ij}\cdot {\hat{\chi }}_{A_{\mathrm{eff}}}\cdot \Delta {\hat{G}}_{j}\right\}\;.$$

Analogue to the derivations for $\Delta M_{i}^{\alpha }$ we turn to a more compact notation upon defining a tensor:

(37) $${\hat{\kappa }}^{\alpha \beta }:=\left(1-\delta^{\alpha \beta }\right){\hat{g}}^{\alpha \beta }-\frac{\Delta {\hat{G}}^{\alpha }}{N}\;,$$

and via introducing here another, quasi mesoscopic, effective susceptibility:

(38) $${\hat{\chi }}_{B_{\mathrm{eff}}}:={\left(\hat{I}+{\hat{\chi }}_{A}\cdot \left(\hat{\nu }-{\hat{G}}_{\mathrm{micro}}-\Delta {\hat{G}}_{\mathrm{micro}}\right)\right)}^{-1}\;\cdot {\hat{\chi }}_{A}\;.$$

We will further see that ${\hat{\chi }}_{B_{\mathrm{eff}}}$ reflects somehow an effective bulk susceptibility of the composite material in the sample. Thus, we may rewrite Equation (34) in similar form as for $\Delta M_{i}^{\alpha }$ in Equation (30):

(39) $$\Delta M^{\alpha }\approx \phi \;{\hat{\chi }}_{B_{\mathrm{eff}}}\cdot \left(\Delta {\hat{G}}^{\alpha }\cdot M+\sum_{\beta }^{N}{\hat{\kappa }}^{\alpha \beta }\cdot \Delta M^{\beta }\right).$$

Again, we end up with a system of linear equations, where apparently $\Delta M^{\alpha }\propto \phi \;{\hat{\chi }}_{B_{\mathrm{eff}}}\cdot \Delta {\hat{G}}^{\alpha }\cdot M$. In the current representation the solution may be again obtained via a direct or iterative procedure. Adopting the notation of generalized matrices the solution is formally computed as:

(40) $$\Delta M^{\alpha }\approx \phi \;\left\{\sum_{\beta }^{N}\left(S^{\alpha \beta }\cdot {\hat{\chi }}_{B_{\mathrm{eff}}}\cdot \Delta {\hat{G}}^{\beta }\right)\right\}\cdot M.$$

Here, the corresponding generalized matrices are defined as:

(41a) $$\begin{array}{cc} {\left[C\right]}^{\alpha \beta } & :={\hat{\chi }}_{B_{\mathrm{eff}}}\cdot {\hat{\kappa }}^{\alpha \beta } \end{array}$$

(41b) $$\begin{array}{cc} S & :={\left(I-\phi C\right)}^{-1}\;. \end{array}$$

Analogue to Equation (33), the S is expanded in a series:

(42) $$S=I+\left\{\sum_{k=1}^{\infty }{\left(\phi C\right)}^{k}\right\}\;.$$

As discussed below, Equation (33), applying our cascading mean-field approach in a first step beyond the leading order we will approximate S≈I. Note that the series in Equation (42) includes the factor ϕ. The volume fraction of magnetic/magnetizable inclusions is usually ϕ≤0.3 for practically relevant MAEs. Thus, we expect the corrections from the series in Equation (42) to be considerably smaller compared to I.

#### 2.3.3. The Average Magnetization

Substituting the above derived expressions for $\Delta M^{\alpha }$ and $\Delta M_{i}^{\alpha }$, resp. ${\langle \Delta M_{i}\rangle }_{S}$, into Equation (25) the average magnetization among all inclusions in the sample, M, becomes:

(43) $$M\approx L\left(A\right)+{\hat{\chi }}_{A}\cdot \left\{\phi^{2}\Delta {\hat{G}}_{\mathrm{macro}}+\Delta {\hat{G}}_{\mathrm{micro}}\right\}\cdot M\;.$$

Here, analogue to Equation (36) we defined a tensor $\Delta {\hat{G}}_{\mathrm{macro}}$ via:

(44) $$\Delta {\hat{G}}_{\mathrm{macro}}:=\frac{1}{N}\sum_{\alpha ,\beta }^{N}\left\{\Delta {\hat{G}}^{\alpha }\;\;\cdot S^{\alpha \beta }\;\cdot {\hat{\chi }}_{B_{\mathrm{eff}}}\;\cdot \Delta {\hat{G}}^{\beta }\right\}\;.$$

Although the relation in Equation (43) effectively represents ‘just’ a system of 3 non-linear equations for the vector M, it is not feasible to solve it directly. Note, especially, that ${\hat{\chi }}_{A}$, ${\hat{\chi }}_{A_{\mathrm{eff}}}$, ${\hat{\chi }}_{B_{\mathrm{eff}}}$, as well as s and S not only depend on A, and thus on M, but are also non-trivially convoluted with each other. Before applying the present approach with some general saturating magnetization behavior we first want to turn to the special case of linear magnetization behavior where most of the principal quantities are decoupled.

### 2.4. The Case of Isotropic Linear Magnetization

In case that we can assume a perfect linear magnetization behavior the present derivation benefits from several simplifications. Most prominently, to yield the magnetic energy in the sample it is fully sufficient to compute only the average magnetization, as we will see below. In contrast, for the general non-linear case the local deviations from the sample mean, or to say the actual magnetization in each point / inclusion, need to be known in order to calculate the magnetic energy of the sample according to Equation (1).

#### 2.4.1. General Relations

In the following, we consider the frequently studied case of an isotropic linear magnetization for each inclusion *a*:

(45) $$M_{a}=\chi \;\hat{I}\cdot H_{a}=\chi H_{a}\;.$$

Then, the magnetic energy in Equation (1) reduces to:

(46) $$U_{\mathrm{mag}}=-\frac{\mu_{0}}{2}V_{S}\phi H_{0}\cdot M\;.$$

We remind that the total amount/volume of magnetizable material in the sample equals $V_{S}\phi$. The average magnetization among all inclusions, M, needs to be calculated self-consistently. Noting, that now ${\hat{\chi }}_{A}=\chi \hat{I}$ and since $\hat{\nu }=\nu \hat{I}$, with $\nu =\frac{1}{3}$ for spherical inclusions, we have:

(47) $${\hat{\chi }}_{A_{\mathrm{eff}}}={\hat{\chi }}_{\mathrm{eff}}=\chi_{\mathrm{eff}}\hat{I}=\frac{\chi }{1+\nu \chi }\hat{I}\;.$$

Accordingly, we may obtain $\Delta {\hat{G}}_{\mathrm{micro}}$ in Equation (36) completely independent of any macroscopic or external parameters like ${\hat{G}}_{\mathrm{macro}}$ or $H_{0}$. The tensor $\Delta {\hat{G}}_{\mathrm{micro}}$ is here, just as it is also the average ${\hat{G}}_{\mathrm{micro}}$ in a general case, an entire and exclusive parameter of the local microstructure. Thus it can be immediately obtained exactly, or to any accurate precision desired.

From Equation (43) with L(A)=χA or, equivalently, from Equation (20) with M=χH we get the exact relation:

(48) $$M=\chi_{\mathrm{eff}}\left\{H_{0}+\left({\hat{G}}_{\mathrm{micro}}+\phi {\hat{G}}_{\mathrm{macro}}+\Delta {\hat{G}}_{\mathrm{micro}}+\phi^{2}\Delta {\hat{G}}_{\mathrm{macro}}\right)\cdot M\right\}\;.$$

Then, we explicitly find:

(49) $$M=\chi_{\mathrm{eff}}{\left\{\hat{I}-\chi_{\mathrm{eff}}\left({\hat{G}}_{\mathrm{micro}}+\phi {\hat{G}}_{\mathrm{macro}}+\Delta {\hat{G}}_{\mathrm{micro}}+\phi^{2}\Delta {\hat{G}}_{\mathrm{macro}}\right)\right\}}^{-1}\;\;\cdot H_{0}\;,$$

and the magnetic energy in the sample is given upon insertion into Equation (46).

This result requires some remarks. Most importantly, all terms in Equation (48), resp. Equation (49), are completely independent of each other, except for $\Delta {\hat{G}}_{\mathrm{macro}}$. Whenever an individual parameter of a given sample changes we may adjust that parameter correspondingly without the need to recalculate the other terms. The only coupling in case of a linear magnetization behavior is entering via $\Delta {\hat{G}}_{\mathrm{macro}}$. The calculation of this tensor is explicitly dependent on the microstructure in the form of the prefactor ${\hat{\chi }}_{B_{\mathrm{eff}}}$ in Equation (44). For linear isotropic magnetization this effective susceptibility reads:

(50) $${\hat{\chi }}_{B_{\mathrm{eff}}}=\chi_{\mathrm{eff}}{\left(\hat{I}-\chi_{\mathrm{eff}}\left({\hat{G}}_{\mathrm{micro}}+\Delta {\hat{G}}_{\mathrm{micro}}\right)\right)}^{-1}\;.$$

If required, for example in microscopic simulation models, the calculation of the local magnetization field $M_{i}^{\alpha }$ in each inclusion can also be formulated yet exactly in the case of a linear magnetization scheme. Substituting Equations (32) and (40) into Equation (10) we find:

(51) $$M_{i}^{\alpha }=\left(\hat{I}+\chi_{\mathrm{eff}}\sum_{j}^{n}s_{ij}\cdot \Delta {\hat{G}}_{j}\right)\cdot \left(\hat{I}+\phi \sum_{\beta }^{N}S^{\alpha \beta }\cdot {\hat{\chi }}_{B_{\mathrm{eff}}}\cdot \Delta {\hat{G}}^{\beta }\right)\cdot M\;.$$

Although it is remarkable that the results, Equations (48)–(51), can be expressed in such a compact and yet exact form for linear magnetization behavior we should be aware of the non-trivial coupling entering via Equation (50). Clearly, a different local arrangement of the particles changes the micro-structural contributions ${\hat{G}}_{\mathrm{micro}}$ and $\Delta {\hat{G}}_{\mathrm{micro}}$. Accordingly, ${\hat{\chi }}_{B_{\mathrm{eff}}}$ also needs to be updated, which requires a recalculation of the macroscopical $\Delta {\hat{G}}_{\mathrm{macro}}$. Similar implications arise in the exact form for the individual $M_{i}^{\alpha }$ in Equation (51).

In addition, in the case of linear magnetization it is therefore worth considering an approximation which allows for a computationally more efficient calculation. Implementing the leading order approximation as introduced around Equation (22) we would simply neglect $\Delta {\hat{G}}_{\mathrm{macro}}$ and $\Delta {\hat{G}}_{\mathrm{micro}}$ in Equation (49) yielding an approximate average magnetization field which we denote $M^{\left(1\right)}$. As suggested above, beyond the leading order we apply s≈I and S≈I in the following. Then, $\Delta {\hat{G}}_{\mathrm{micro}}$ in Equation (36) is estimated as:

(52) $$\Delta {\hat{G}}_{\mathrm{micro}}\approx \frac{\chi_{\mathrm{eff}}}{n}\sum_{i}^{n}{\left(\Delta {\hat{G}}_{i}\right)}^{2}\;.$$

Accordingly, we obtain an approximation for ${\hat{\chi }}_{B_{\mathrm{eff}}}$ in Equation (50) and the local magnetization fields $M_{i}^{\alpha }$ in Equation (51) are approximated via:

(53) $$M_{i}^{\alpha }\approx \left(\hat{I}+\chi_{\mathrm{eff}}\Delta {\hat{G}}_{i}\right)\cdot \left(\hat{I}+\phi \;{\hat{\chi }}_{B_{\mathrm{eff}}}\cdot \Delta {\hat{G}}^{\alpha }\right)\cdot M^{\left(1\right)}\;.$$

In addition, we can estimate $\Delta {\hat{G}}_{\mathrm{macro}}$ because with S≈I Equation (44) reduces to:

(54) $$\Delta {\hat{G}}_{\mathrm{macro}}\approx \frac{\chi_{\mathrm{eff}}}{N}\sum_{\alpha }^{N}\left(\Delta {\hat{G}}^{\alpha }\cdot {\hat{\chi }}_{B_{\mathrm{eff}}}\cdot \Delta {\hat{G}}^{\alpha }\right)\;.$$

Furthermore, Equations (52) and (54) can be inserted in Equation (49) yielding an improved average magnetization. Whether, or how, to approximate ${\hat{\chi }}_{B_{\mathrm{eff}}}$, resp. $\Delta {\hat{G}}_{\mathrm{micro}}$ and $\Delta {\hat{G}}_{\mathrm{macro}}$, depends on the characteristics of the actual MAE sample, the desired degree of precision, and the available computation resources.

#### 2.4.2. Alternative Formulation of the Role of the Effective Mesoscopic Susceptibility

An alternative way for consideration of the microstructure contribution will become very enlightening here. We consider an isotropic linear magnetization scheme in which Equations (24) and (32) are exact with L(A)=χA and $\nabla_{A}L\left(A\right)=\chi \hat{I}$. With Equation (22) and the definition of $\Delta {\hat{G}}_{\mathrm{micro}}$ in Equation (36) we can rewrite Equation (24):

(55) $$M^{\alpha }=\chi \left\{H_{0}+\phi {\hat{G}}^{\alpha }\cdot M+\phi \sum_{\beta \neq \alpha }^{N}{\hat{g}}^{\alpha \beta }\cdot \Delta M^{\beta }+\left({\hat{G}}_{\mathrm{micro}}+\Delta {\hat{G}}_{\mathrm{micro}}-\hat{\nu }\right)\cdot M^{\alpha }\right\}\;.$$

Using here the definition in Equation (14) as well as that of ${\hat{\chi }}_{B_{\mathrm{eff}}}$ for isotropic linear magnetization in Equation (50), we finally arrive at:

(56) $$M^{\alpha }={\hat{\chi }}_{B_{\mathrm{eff}}}\cdot \left\{H_{0}+\phi \;\sum_{\beta \neq \alpha }^{N}{\hat{g}}^{\alpha \beta }\cdot M^{\beta }\right\}\;.$$

This is a quite compact and remarkable result. Although it is exclusively determined for the case of an (isotropic) linear magnetization behavior, it clearly shines a bright light on the interpretation of effective susceptibility ${\hat{\chi }}_{B_{\mathrm{eff}}}$. Apparently, Equation (56) exactly represents the dipole calculation scheme of the magnetization distribution within a body containing a volume fraction ϕ of magnetizable material, which obeys itself a bulk magnetization behavior of the composite as reflected by the susceptibility ${\hat{\chi }}_{B_{\mathrm{eff}}}$. Generally, and depending on the actual microscopic particle distribution, this new effective bulk susceptibility ${\hat{\chi }}_{B_{\mathrm{eff}}}$ is anisotropic, although the original bulk susceptibility of the inclusion material is isotropic $\hat{\chi }=\chi \hat{I}$. Accordingly, once the ${\hat{\chi }}_{B_{\mathrm{eff}}}$ for a given microstructure, via calculating ${\hat{G}}_{\mathrm{micro}}$ and $\Delta {\hat{G}}_{\mathrm{micro}}$, is known, it is also possible to apply alternative solution approaches like well-established Finite-Element Methods for an anisotropic magnetization behavior in a given sample body. The form of the microstructure determines the effective magnetization behavior on meso-, resp. macroscale, just like it does $\chi_{\mathrm{eff}}=\chi /\left(1+\chi \nu \right)$ on a particle scale. For an isotropic [33], as well as for an ‘isotropic-like’ [32,34], particle distribution the micro-contributions vanish, ${\hat{G}}_{\mathrm{micro}}\to 0$ and $\Delta {\hat{G}}_{\mathrm{micro}}\to 0$, and thus ${\hat{\chi }}_{B_{\mathrm{eff}}}=\chi_{\mathrm{eff}}\hat{I}$. An anisotropic microstructure likewise gives rise to an anisotropic ${\hat{\chi }}_{B_{\mathrm{eff}}}$.

Beside a better understanding of how the different length scales in an MAE may be separated and at the same time also influence each other, the important message here is how alternative, and computationally advantageous, approaches can enter the formalism to obtain a comprehensive, detailed, and accurate, but at the same time effort- or complexity-reduced description of the magnetization fields in an MAE sample. The above-derived relations and interpretations concern the case of a linear magnetization behavior. In the following we want to consider the corresponding implications for the general case of a more realistic magnetization scheme.

### 2.5. Cascading Approximation Scheme for Saturating Magnetization

To be able to provide an accurate approximate description of the magnetization fields in the case of non-linear magnetization behavior, it is necessary to adopt a cascading scheme and start with the leading order in the form:

(57) $$M^{\left(1\right)}=L\left(A(M^{\left(1\right)})\right)=L\left(H_{0}+\left(\phi {\hat{G}}_{\mathrm{macro}}+{\hat{G}}_{\mathrm{micro}}-\hat{\nu }\right)\cdot M^{\left(1\right)}\right)\;.$$

For some given composition, i.e., microstructure, sample shape or volume fraction of magnetizable particles, all terms in Equation (57) are computed independently. In the simplest, or leading order, we may assign each particle in the sample this average magnetization $M^{\left(1\right)}$ as obtained upon solving just a single 3-dimensional equation, i.e., Equation (57). Beyond the simple leading order ($M_{i}^{\alpha }\approx M^{\left(1\right)},\;\forall i,\alpha$) we consider the following procedure.

From the solution of Equation (57) we not only find a first approximation for the average magnetization among all particles, but also an approximation for the average magnetic field A. Using Equations (28) and (29) we obtain the tensors ${\hat{\chi }}_{A}$ and ${\hat{\chi }}_{A_{\mathrm{eff}}}$. Furthermore, one can then proceed to calculate ${\hat{\chi }}_{B_{\mathrm{eff}}}$ via Equations (36) and (38). Analogously to the case of linear magnetization in Section 2.3 we use s≈I and S≈I. The local magnetization $M_{i}^{\alpha }$ of an individual particle is then evaluated in the next approximation step as:

(58) $$M_{i}^{\alpha }\approx \left(\hat{I}+{\hat{\chi }}_{A_{\mathrm{eff}}}\Delta {\hat{G}}_{i}\right)\cdot \left(\hat{I}+\phi \;{\hat{\chi }}_{B_{\mathrm{eff}}}\cdot \Delta {\hat{G}}^{\alpha }\right)\cdot M^{\left(1\right)}\;.$$

This result is similar to the approximation in the case of a linear magnetization behavior in Equation (53). Note however a small difference, the tensorial representation of ${\hat{\chi }}_{A_{\mathrm{eff}}}$, since it may become anisotropic in the case of a saturating magnetization behavior.

We want to highlight that the present approximation approach is completely determined if the tensors ${\hat{G}}^{\alpha }$ and ${\hat{G}}_{i}$ are evaluated for a given sample form and microstructure. From these, one directly derives the averages ${\hat{G}}_{\mathrm{macro}}$ and ${\hat{G}}_{\mathrm{micro}}$, as well as the deviations $\Delta {\hat{G}}^{\alpha }$ and $\Delta {\hat{G}}_{i}$. With Equation (57) the calculation of magnetization fields requires only one single three dimensional self-consistent equation. The rest of the computation are straightforward addition and multiplication functions.

In the following we will work out the here developed approximation scheme for some examples to demonstrate its applicability in practice.

## 3. Application to Selected Examples

In this section we want to apply the present approach to some example calculations. In the first situation we consider the distribution of a finite number *n* of particles obeying some specific saturating magnetization behavior under the application of an external field $H_{0}$. We compare the approximation results to the exact computation of the 3n×3n system of self-consistent non-linear equations. In a second example we derive tensors ${\hat{G}}_{\mathrm{macro}}$ and $\Delta {\hat{G}}^{\alpha }$ for the meso- and macroscopic effects for a sample of cylindrical form. To prove the accuracy of our approach against an exact calculation for an entire macroscopic sample with, at the same time, a fully resolved particle structure on a local scale in 3 dimensions would require a tremendous computational effort for the ‘true’ or precise description. For our approximation scheme such calculation is straightly feasible upon combining the subsequent example schemes for micro- and macroscale in Equations (57) and (58). We may set up a 2-dimensional model in the future where an exact calculation of $∼O(10^{6-7})$ particles defining a 2D quasi-macroscopic sample is feasible.

### 3.1. Microstructure Calculation for Finite Number of Inclusions

To compare exact results from solving the full non-linear set of equations self-consistently and the approximation from the here developed approach for an actual particle configuration we introduce the following properties. We characterize the magnetization behavior of the particles via an isotropic saturating magnetization of the form:

(59) $$L\left(H\right)=L\left(H\right)\frac{H(r)}{\left|H(r)\right|}=M_{s}\left(\coth\left(\zeta H\right)-\frac{1}{\zeta H}\right)\frac{H(r)}{\left|H(r)\right|}\;.$$

**Figure 3:**
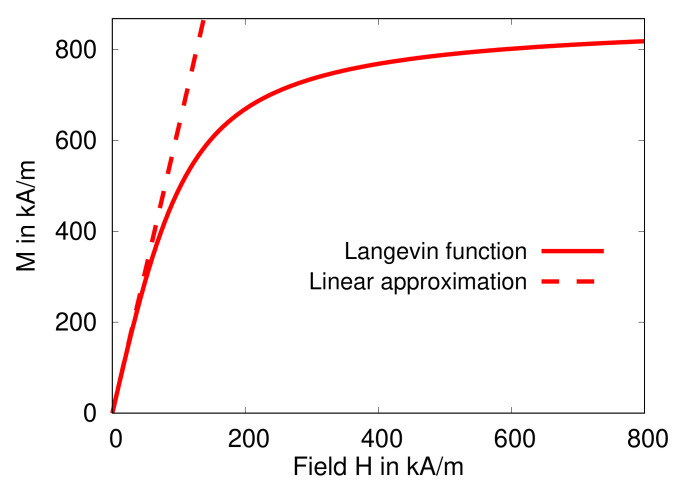
A plot of the Langevin-type saturating magnetization function in Equation (59) with $M_{\mathrm{sat}}=868\;\mathrm{kA}/m$ and ζ=0.0218m/kA. Additionally, the linear approximation in the low field limit is displayed, i.e., M=χH with $\chi =\frac{\zeta M_{\mathrm{sat}}}{3}\approx 6.31$.

This function represents a Langevin-type behavior, and using the parameters $M_{\mathrm{sat}}=868\;\mathrm{kA}/m$ and ζ=0.0218m/kA it was shown to adequately reproduce the magnetization behavior of micron-sized carbonyl iron particles [39,40]. The function in Equation (59) is plotted in Figure 3 together with its linear approximation, i.e., M=χH in the limit H→0. Apparently, the linear approximation is only adequate as far as H<100kA/m. Hence, it is crucial to account for saturating effects and consider a more realistic form of the magnetization behavior, i.e., beyond the linear approximation, to reasonably model authentic experimental situations. Nevertheless, because of its great computation benefits and to obtain a first approximate description, the linear magnetization assumption represents a substantial modeling strategy in theory [16,32,33,44,48,49,55].

Upon solving a system of non-linear equations of the form as presented in Equations (2)–(4) the exact magnetization field within the dipole model is found. In the following we consider some selected particle arrangements of a small number *n* of identical spherical inclusions, with the same particle volume *v* and magnetization property according to Equation (59). Under the application of an external field $H_{0}$, the induced magnetization field $M_{i}$ in particle *i* located at $r_{i}$ is given through:

(60) $$M_{i}=L\left(H_{0}-\frac{1}{3}M_{i}+\frac{v}{4\pi }\sum_{j\neq i}^{n}\frac{3\left(r_{ij}\cdot M_{j}\right)\;r_{ij}-r_{ij}^{2}\;M_{j}}{r_{ij}^{5}}\right)\;.$$

This system of 3n×3n equations we solve via the Newton–Raphson technique [56]. The average of these individual magnetization fields $M_{i}$ over all *n* particles defines the exact average magnetization:

(61) $$M=\frac{1}{n}\sum_{i}^{n}M_{i}\;.$$

The application of the approximation scheme to a finite number of particles is straightforward. First, we obtain the ${\hat{G}}_{i}$∀i∈n according to Equation (13) and calculate its average ${\hat{G}}_{\mathrm{micro}}$ via Equation (15). The leading order approximation Equation (57) with pure microstructure effect is then calculated via the single 3-dimensional self-consistent equation:

(62) $$M^{\left(1\right)}=L\left(H_{0}-\frac{1}{3}M^{\left(1\right)}+{\hat{G}}_{\mathrm{micro}}\cdot M^{\left(1\right)}\right)\;.$$

With $\Delta {\hat{G}}_{i}={\hat{G}}_{i}-{\hat{G}}_{\mathrm{macro}}$, the magnetization field $M_{i}$ in each particle *i* beyond the leading order is adopted from Equation (58) and approximated as:

(63) $$M_{i}\approx M_{i}^{\left(1\right)}=\left(\hat{I}+{\hat{\chi }}_{A_{\mathrm{eff}}}\cdot \Delta {\hat{G}}_{i}\right)\cdot M^{\left(1\right)}\;.$$

The effective susceptibility tensor ${\hat{\chi }}_{A_{\mathrm{eff}}}$ results from Equations (28) and (29) with $A=H_{0}-\frac{1}{3}M^{\left(1\right)}+{\hat{G}}_{\mathrm{micro}}\cdot M^{\left(1\right)}$. In the present case of an isotropic saturating magnetization, the susceptibility tensor ${\hat{\chi }}_{A}$ is obtained via:

(64) $${\hat{\chi }}_{A}=\nabla L\left(A\right)=\frac{L(A)}{A}\hat{I}+\left(L^{\prime }\left(A\right)-\frac{L(A)}{A}\right)e_{A}e_{A}\;.$$

We compare the solution of Equation (60) for all i∈n to our approximation approach Equation (63). In order to quantify the precision of our scheme, we use the relative absolute error $\delta M_{i}$ in the following form:

(65) $$\delta M_{i}=\frac{\left|M_{i}^{\left(1\right)}-M_{i}\right|}{\left|M_{i}\right|}\;,$$

and find both, the maximum, $\max\left\{\delta M_{i},i\in n\right\}$, and the average error among all particles:

(66) $$\langle \delta M_{i}\rangle =\frac{1}{n}\sum_{i}^{n}\delta M_{i}\;.$$

**Figure 4:**
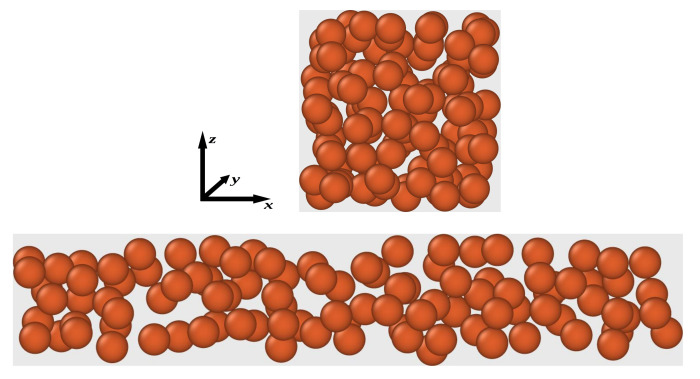
Visualization of the particle arrangements considered here. (**Top**) Randomly generated microstructure within a cubic cell. (**Bottom**) Randomly generated microstructure within an elongated cylinder with its main axis along *x*-direction. The visualization is preformed using OVITO [57].

For example, in the case of a simulation or any other explicit treatment of an MAE sample it is necessary to describe the magnetization fields in the corresponding many-particle system. Here, we want to consider a system of 100 particles arranged randomly at a volume fraction ϕ=0.3 inside some fictitious boundaries assuring that the particles do not overlap each other. In the first case the sample boundaries are specified by a cube, and, thus, it should more or less represent a part of an isotropic microstructure. In the second case we set the boundaries to an elongated cylinder imitating a part of a rather chain-like particle structure. The main axis of the cylinder is aligned with the *x*-axis. The randomly generated particle configurations in both cases are visualized in Figure 4. In the following the diameter of one spherical particle defines the unit length in the system.

In the first case, see top of Figure 4, we set each side of the boundaries an equal length of $l_{x}=l_{y}=l_{z}\approx 5.59$. Accordingly, the volume fraction of magnetizable particles in the cell becomes ϕ≈0.3. For the arrangement shown in the top of Figure 4 we find the following average microstructure tensor expressed in Cartesian coordinates:

(67) $${\hat{G}}_{\mathrm{micro}}=\left(\begin{array}{ccc} 8.91\times 10^{-5} & -2.65\times 10^{-3} & -1.81\times 10^{-3}\\ -2.65\times 10^{-3} & -1.8\times 10^{-3} & -5.06\times 10^{-4}\\ -1.81\times 10^{-3} & -5.06\times 10^{-4} & 1.72\times 10^{-3} \end{array}\right)\;.$$

Using this ${\hat{G}}_{\mathrm{micro}}$ in Equation (62), we find an approximation for the average magnetization among all particles, $M^{\left(1\right)}$. The exact magnetization fields are calculated according to Equation (60). In Table 1 we compare precise and approximate calculations. Exemplary, we apply two different external fields $H_{0}$. In the first line in Table 1 we summarize the results for a moderately low $H_{0}$ aligned along the *x*-axis. In the second line, a moderately large field inclined with respect to the cubic cell is applied.

The comparison between exact M and approximate $M^{\left(1\right)}$ shows a quite reasonable agreement for both $H_{0}$. In addition to it, both the average magnetization is obtained in good accordance and the individual magnetization field within each particle is found to be rather accurate. On average the deviations of the approximate $M_{i}^{\left(1\right)}$ from the precise $M_{i}$ are less than 1%. The maximal error here is found to be just ≲2.2%. In the Supplementary Materials we provide a full list of all $M_{i}$ and $M_{i}^{\left(1\right)}$ (i∈[1,100]) to demonstrate the remarkable accuracy of our approximation scheme. We highlight that this high degree of precision is achieved already in the quite simple form provided via Equations (62) and (63). The computation benefit compared to the full solution in Equation (60) on the other hand is striking as the approximation only requires the self-consistent solution of a single 3-dimensional equation.

Let us consider now the case where the particles are arranged inside a small but elongated cylinder with its main axis aligned along the *x*-direction. The aspect ratio Γ of length to diameter is chosen to be Γ=6 and the volume is adjusted such that the n=100 spherical particles occupy again a volume fraction of ϕ≈0.3. The particle structure is visualized in the bottom of Figure 4. The tensor ${\hat{G}}_{\mathrm{micro}}$ we found for this case reads:

(68) $${\hat{G}}_{\mathrm{micro}}=\left(\begin{array}{ccc} 5.73\times 10^{-2} & 1.57\times 10^{-3} & -3.64\times 10^{-3}\\ 1.57\times 10^{-3} & -3.01\times 10^{-2} & -4.42\times 10^{-3}\\ -3.64\times 10^{-3} & -4.42\times 10^{-3} & -2.72\times 10^{-2} \end{array}\right)\;.$$

Apparently, and in contrast to the cubic cell in Equation (67), the tensor in Equation (68) exhibits much larger absolute values in the diagonal elements. This property reveals the rather anisotropic structure compared to the cubic boundaries. Clearly, $G_{{\mathrm{micro}}_{xx}}$ adopts the largest value here because the elongated structure is aligned along *x*.

Please note the following: Ideally, for an isotropic distribution of particles inside cubic boundaries ${\hat{G}}_{\mathrm{micro}}$ vanishes. When averaging over several randomly generated particle distributions inside a cubic cell, all entries of ${\hat{G}}_{\mathrm{micro}}$ in Equation (67) equally diminish. In contrast, averaging over several randomly generated distributions inside elongated cylindrical boundaries result in only the non-diagonal elements in Equation (68) to vanish, whereas the diagonal elements converge towards a finite value of $O(10^{-2})$. Since ${\hat{G}}_{\mathrm{micro}}$ is traceless and due to cylindrical symmetry then $G_{{\mathrm{micro}}_{yy}}=G_{{\mathrm{micro}}_{zz}}=-\frac{1}{2}G_{{\mathrm{micro}}_{xx}}$ on average. In addition, with increasing particle number *n* the absolute values of all elements in Equation (67), resp. the non-diagonal elements in Equation (68), diminish. For a single, explicit structure of n=100 particles we usually find that such ‘negligible’ elements are of order $O(10^{-3})$ in maximum absolute value.

Again, upon using Equation (68) in Equation (62) we find an approximation for the average magnetization among all particles, $M^{\left(1\right)}$ and the individual $M_{i}^{\left(1\right)}$ are found via Equation (63). The precise solution is calculated according to Equation (60). We consider the same two $H_{0}$ as in the case of cubic boundaries. In Table 2 we compare the corresponding results.

Again, we find very reasonable agreement between the full and the approximate calculation. On average, the absolute deviations $\langle \delta M_{i}\rangle$ are clearly below 1%. The maximum error found for an individual particle is just ∼1.44%. The full list of all $M_{i}$ and $M_{i}^{\left(1\right)}$ is provided in the Supplementary Materials. Clearly, upon comparing the results in Table 1 and Table 2, we note that upon applying identical $H_{0}$ the particles arranged in an elongated structure along the *x*-axis feature considerably larger components of the magnetization field in *x*-directions as compared to particles arranged inside a cubic cell.

### 3.2. Macroscopic Shape Effect for a Cylindrical Sample

According to our approach the relevant quantity describing the effect on the magnetization field due to the macroscopic shape of a sample is given by the tensorial operator defined in Equation (14). There, for the sake of consistency with the microscopic operator in Equation (13), it is expressed in a discrete form. Formally, Equation (14) represents the discretization of the volume integration over the entire sample body. With Equation (7) in Equation (14) and the number of volume elements, N→∞ the mesoscopic volume elements become infinitesimal, $V^{\beta }\to dV$. Thus, turning to a continuous representation α→r we have to calculate the following integral equation:

(69) $$\hat{G}\left(r\right)=\frac{1}{4\pi }\int_{V_{C}}d^{3}r^{\prime }\frac{3\left(r^{\prime }-r\right)\left(r^{\prime }-r\right)-{\left|r^{\prime }-r\right|}^{2}}{{\left|r^{\prime }-r\right|}^{5}}\Theta \left(\left|r^{\prime }-r\right|\right)\;.$$

**Figure 5:**
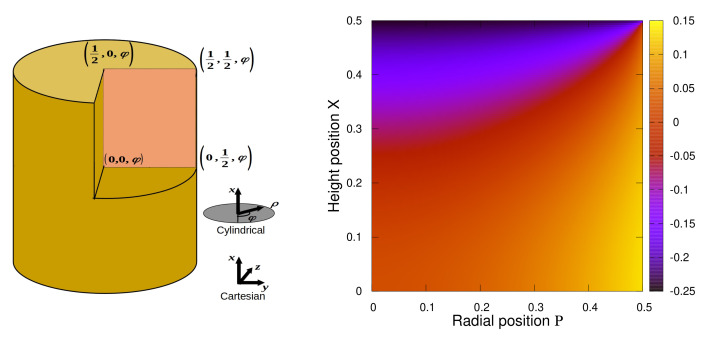
(**Left**) Sketch of the coordinate system used to display the results. We use cylindrical coordinates (x,ρ,φ) with $\left(x,\rho =\sqrt{y^{2}+z^{2}},\varphi =\arcsin\left(\frac{y}{\rho }\right)\right)$. The origin is located in the center of mass of the cylinder. In reduced units $X=\frac{x}{L}$ and $P=\frac{\rho }{D}$ accordingly $X\in [-\frac{1}{2},\frac{1}{2}]$, $P\in [0,\frac{1}{2}]$ and φ∈[0,2π). (**Right**) Plotting $\Delta G_{xx}\left(X,P\right)$ for a cylinder with aspect ratio $\Gamma =\frac{L}{D}=1$.

Function Θ(r) denotes the Heavyside function, which is a defined unity for r>0 and zero otherwise, in order to omit the pole at $r=r^{\prime }$, i.e., α≠β in Equation (14). The integration in Equation (69) is taken over the volume of the cylindrical sample $V_{C}$ and shall be performed for each component of $\hat{G}$. In the following we denote the symmetry, or main, axis of the cylinder as the *x*-direction and the perpendicular circular plane shall be generated by *y* and *z*, see Figure 5. The origin of the coordinate system is defined as the center of the cylinder’s mass.

Due to cylindrical symmetry of the sample and the form of the integrand in Equation (69), e.g., $\hat{G}$ is symmetric with $tr(\hat{G})=0$, one can conclude that $G_{yy}=G_{zz}=-\frac{1}{2}G_{xx}$ and $G_{yz}=G_{zy}=0$. Furthermore, the two remaining cross-terms $G_{xy}=G_{yx}$ and $G_{xz}=G_{zx}$ must be equivalently centrosymmetric with respect to the cylinder main axis. Thus, expressed in Cartesian coordinates we find:

(70) $$\hat{G}=\left(\begin{array}{ccc} G_{xx} & G_{x\rho }\frac{y}{\rho } & G_{x\rho }\frac{z}{\rho }\\ G_{x\rho }\frac{y}{\rho } & -\frac{G_{xx}}{2} & 0\\ G_{x\rho }\frac{z}{\rho } & 0 & -\frac{G_{xx}}{2} \end{array}\right)\;.$$

The cross-term we denote here $G_{x\rho }$ as it describes the interrelation between the symmetry axis along $e_{x}$ and the lateral direction along $e_{\rho }$, with $\rho =\sqrt{y^{2}+z^{2}}$. Finally, due to symmetry considerations $G_{xx}$ and $G_{x\rho }$ can only depend on the ‘height’ position *x* and the lateral coordinate ρ. The functions $G_{xx}\left(x,\rho \right)$ and $G_{x\rho }\left(x,\rho \right)$ are given in Appendix A.

The tensor ${\hat{G}}_{\mathrm{macro}}$ is found upon averaging $\hat{G}$ over the sample volume:

(71) $${\hat{G}}_{\mathrm{macro}}=\frac{1}{V_{C}}\int_{V_{C}}d^{3}r\;\;\hat{G}\;.$$

Apparently, the cross-terms immediately vanish due to the centrosymmetric form and we find:

(72) $${\hat{G}}_{\mathrm{macro}}=\left(\begin{array}{ccc} \langle f_{\mathrm{macro}}\rangle & 0 & 0\\ 0 & -\frac{\langle f_{\mathrm{macro}}\rangle }{2} & 0\\ 0 & 0 & -\frac{\langle f_{\mathrm{macro}}\rangle }{2} \end{array}\right)\;.$$

Here, $\langle f_{\mathrm{macro}}\rangle$ describes the volume average of $G_{xx}$ according to Equation (71). We denote the height, or length, of the cylindrical sample as *L* and its cross section diameter as *D*. Then, for being a scale-free quantity, $\langle f_{\mathrm{macro}}\rangle$ can only be a single parametric function of the aspect ratio $\Gamma =\frac{L}{D}$. In Ref. [36] we plotted the dependency $\langle f_{\mathrm{macro}}\rangle \left(\Gamma \right)$ for a cylinder in comparison to the corresponding results for a spheroid and a rectangular rod. There, we also show that for ϕ→1, i.e., hypothetically pure magnetizable material, we obtain the relation $1/3-\langle f_{\mathrm{macro}}\rangle =N_{\|}$, with $N_{\|}$ the demagnetization factor of a bulk magnetic cylinder homogeneously magnetized along its main axis. The function $N_{\|}\left(\Gamma \right)=1/3-\langle f_{\mathrm{macro}}\rangle \left(\Gamma \right)$ we derive by the present method exactly resembles previously reported findings [58,59]. In addition to the compliance with the demagnetizing factor, our here derived formalism features a great flexibility in predicting the magnetization field inside a composite sample in an approximation description.

Let us shortly consider the approximation scheme introduced in Section 2.5. With some prescribed microstructure, i.e., ${\hat{G}}_{\mathrm{micro}}$ in the compound MAE and given ${\hat{G}}_{\mathrm{macro}}$, e.g., Equation (72) we immediately may predict the average magnetization among all magnetizable inclusions in approximate, but general form via Equation (57). In the most elementary case of a homogeneous-like, or random isotropic, particle structure we have simply ${\hat{G}}_{\mathrm{micro}}=\hat{0}$ [33,34,52]. Neglecting the microscopic contribution, Equation (57) characterizes primarily the sample shape effect:

(73) $$M^{\left(1\right)}=L\left(H_{0}+\left(\phi {\hat{G}}_{\mathrm{macro}}-\hat{\nu }\right)\cdot M^{\left(1\right)}\right)\;.$$

Just via the solution of this 3-dimensional equation we find a first approximation for the average magnetization field $M\approx M^{\left(1\right)}$ without the necessity to reconsider the full sample if the magnetization property L(H), the applied field $H_{0}$, or the amount ϕ of inclusion particles is modified. Furthermore, in the case of a homogeneous-like, or random isotropic, particle structure Equation (58) is adopted to read:

(74) $$M^{\left(1\right)}\left(r\right)=\left(\hat{I}+\phi {\hat{\chi }}_{A_{\mathrm{eff}}}\cdot \Delta \hat{G}\left(r\right)\right)\cdot M^{\left(1\right)}\;.$$

**Figure 6:**
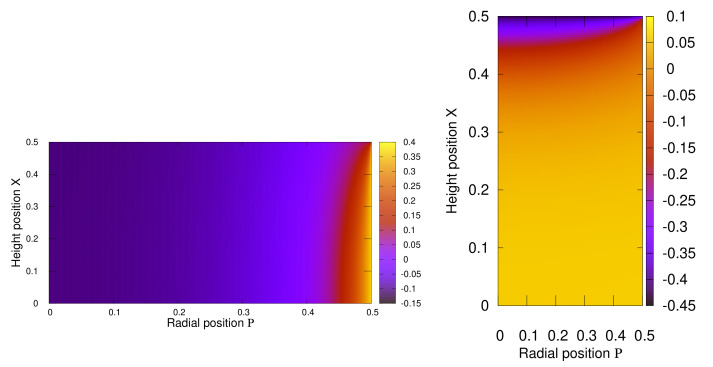
The function $\Delta G_{xx}\left(X,P\right)$ for a cylinder with aspect ratio Γ=0.1 (**Left**) and Γ=5 (**Right**). To illustrate the oblate, resp. prolate, form of the cylinders we stretched the axes.

Thus, we immediately note that $\Delta \hat{G}\left(r\right)=\hat{G}\left(r\right)-{\hat{G}}_{\mathrm{macro}}$, describes the mesoscopic deviations in the magnetization field due to the macroscopic shape effects. In Figure 5 and Figure 6 we display our results for the diagonal component $\Delta G_{xx}=G_{xx}-\langle f_{\mathrm{macro}}\rangle$ for different aspect ratios of the cylinder (Γ=1.0, Γ=0.1, and Γ=5.0).

Due to symmetry considerations it is obvious that $\Delta G_{xx}=\Delta G_{xx}\left(x,\rho \right)$ can only depend on the height position *x* and the radial position ρ. We introduce dimensionless units $X=\frac{x}{L}$ and $P=\frac{\rho }{D}$, to represent $\Delta G_{xx}\left(X,P\right)$ with $X\in [-\frac{1}{2},\frac{1}{2}]$ and $P\in [0,\frac{1}{2}]$. The angular coordinate $\varphi =\arcsin(\frac{y}{\rho })$ with φ∈[0,2π) is not relevant. Furthermore, since from Equation (69) we note the symmetry x→−x for $G_{xx}$ we also find $\Delta G_{xx}\left(-X,P\right)=\Delta G_{xx}\left(X,P\right)$ and display our results for the cut $X\in [0,\frac{1}{2}]$ and $P\in [0,\frac{1}{2}]$, see left sketch in Figure 5. On the right in Figure 5, $\Delta G_{xx}\left(X,P\right)$ is plotted via a 2-dimensional color map for a symmetric cylinder, i.e., Γ=1. It clearly indicates, that by applying an external field $H_{0}$ along the *x*-direction, the magnetization field into the same direction *x* (accordingly $G_{xx}$) is reduced at the top, and correspondingly also at the bottom, of the cylinder compared to the average magnetization field. In contrast, at the lateral, or outer, fringe the magnetization field is enhanced compared to the average magnetization. These variations are due to the specific cylindrical form of the sample.

In Figure 6 the corresponding $\Delta G_{xx}\left(X,P\right)$ for an oblate (Γ=0.1) and a prolate (Γ=5) cylinder are shown. The results are somehow similar to the case Γ=1, but also a systematic difference is visible. Clearly, in the case of an oblate cylinder the region with reduced magnetization (compared to the average M) is largely grown. For Γ=0.1 it spans an entire part inside the sample (for all positions with p<0.39) from the top to the bottom. The reduction effect in that region is relatively small ($G_{xx}\approx -0.1$). In contrast, the part in the lateral fringe, where an enhancement effect occurs is reduced in size but the enhancement effect itself is increased instead ($G_{xx}\approx 0.35$). In the case of a prolate cylinder, with Γ=5, the situation is exactly reversed. Here, the enhancement region is clearly widened (spanning through the entire cylinder where X∈[−0.34,0.34]), but quantitatively weakened. And the reduction effect is now of a large absolute magnitude, but concentrated to a small slice on the top (and the bottom) of the cylinder.

**Figure 7:**
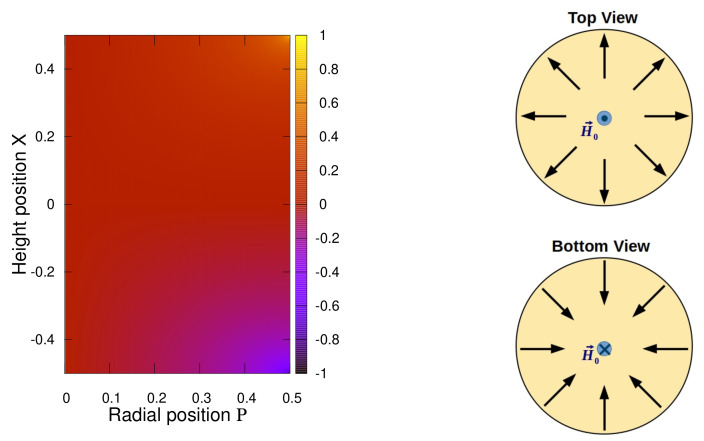
(**Left**) The function $\Delta G_{x\rho }\left(X,P\right)=G_{x\rho }\left(X,P\right)$ for a cylinder with aspect ratio Γ=1. Note, to visualize the property $\Delta G_{x\rho }\left(-X,P\right)=-\Delta G_{x\rho }\left(X,P\right)$ we plot here the full cylinder length $X\in [-\frac{1}{2},\frac{1}{2}]$. (**Right**) Illustration of the central symmetry of the cross terms in Equation (70). We sketch the lateral magnetization (in *y*-*z*-plane) on the top and bottom of the cylinder upon applying an external field $H_{0}$ along the positive *x*-direction.

Regarding the cross-term $\Delta G_{x\rho }\left(X,P\right)=G_{x\rho }\left(X,P\right)$, we plot the corresponding 2-dimensional color map for a symmetric cylinder Γ=1 on the left in Figure 7. Since this function is antisymmetric with respect to X→−X the full length of the cylinder is displayed here. Obviously, in the largest part of the cylindrical body the cross-term nearly vanishes. Only right at the edges does it adopt quite large absolute values, positive on the top ($x∼\frac{L}{2}$) and negative on the bottom ($x∼-\frac{L}{2}$). In fact, due to the perfectly shaped edge in the geometrical representation of a cylinder we encounter a discontinuity exactly at $\rho =\frac{D}{2}$ and $x=\pm \frac{L}{2}$. Any real body can not obey such mathematically shaped edges and thus we consider such discontinuity as a modeling artifact here. Nevertheless, the discontinuity is fortunately only of a logarithmic order and we expect our function $G_{x\rho }$ to be yet reasonable if $\rho <\frac{D}{2}$ or $\left|x\right|<\frac{L}{2}$. In order to give an idea about the ‘full’ centrosymmetric property of the cross-terms in Equation (70) we sketch it in vectorial form $G_{x\rho }\frac{ye_{y}+ze_{z}}{y^{2}+z^{2}}$ on the right in Figure 7. The application of an external field $H_{0}$ along the positive *x*-direction imposes, additionally to the magnetization field along *x*, as well as components in the lateral direction. At the edge on the bottom of the cylinder this lateral magnetization field is oriented ‘inwardly’ to the main axis. In contrast, on the top of the cylinder it is oriented ‘outwardly’ to the edge.

**Figure 8:**
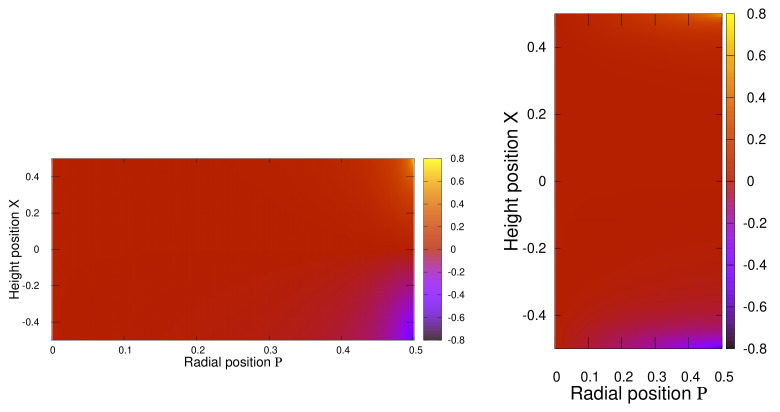
The function $\Delta G_{x\rho }\left(X,P\right)$ for a cylinder with aspect ratio Γ=0.1 (**Left**) and Γ=5 (**Right**). To illustrate the oblate, resp. prolate, form of the cylinders we stretched the axes.

For completeness, we plot in Figure 8 the analogue results for $\Delta G_{x\rho }\left(X,P\right)$ in the case of an oblate cylinder, Γ=0.1 on the left, and a prolate cylinder, Γ=5 on the right. Apparently, the cross-term is almost identical for variations of the cylinder aspect ratio. Yet one systematic difference may be identified. In an oblate cylinder the major contribution to $\Delta G_{x\rho }\left(X,P\right)$ is found at the edges along the *x*-axis. In prolate cylinders, the principal part exists at the edges along the radial axis.

## 4. Conclusions

To understand the behavior of an MAE sample it is vital to capture the full extent of the magnetic interactions, which drive the changes of material properties under the application of an external magnetic field. From fundamental magneto-statics one immediately notes that these interactions are energetically equivalent on different length scales. Consequently, any comprehensive description of MAE samples is sophisticated as several orders of magnitude in length scale must be bridged. In the present work we developed an approximation approach which allows to greatly decouple the short- and long-range effects to the magnetization field in MAE composites. This is achieved upon introducing a Cascading Mean-Field scheme where we split the magnetization of an individual particle into three contributions: The macroscopic average magnetization field among all particles in the sample, the deviations due to the mesoscopic position within the sample volume, and the deviations resulting from the microscopic location with respect to the surrounding particle structure. In order to cover practically relevant situations, the derivation is performed in a possibly general manner. The intrinsic magnetization behavior of the inclusion material, the shape of the macroscopic sample, the actual form of the local microstructure, the overall amount of the inclusion particles, as well as the externally applied field can be adjusted according to the actual conditions without the need to restart the full computation all over again. This flexibility exemplifies the potential of our Cascading Mean-Field approach.

As a demonstration example for the mesoscopically varying magnetization fields due to a non-ellipsoidal shape of a macroscopic sample we considered a cylindrical form. Our results reveal that deviations from the macroscopic average are found most notably near the sample boundaries and strongly depend on the aspect ratio of the cylinder, see Figure 6. In particular, the cross-term $\Delta G_{x\rho }$, coupling lateral to parallel components, displays quite large values, up to order unity near the sample edges, see Figure 7. Thus, for a sample with volume fraction ϕ of magnetizable particles, the relative mesoscopic deviations from the macroscopic magnetization average can reach O(ϕ). In order to illustrate the application of our approach for a resolved particle microstructure we considered an ensemble of 100 inclusions and compared the approximation result to the full self-consistent solution of the problem. These comparisons, as summarized in Table 1 and Table 2, clearly demonstrate the high accuracy of our approach and the relative errors usually range in the order of just ∼1%.

The explicit particle arrangements are usually considered in computer simulations [37,40,45,46,47,48,49,53,60,61,62], where it is essential to calculate the magnetization fields in individual particles to account for the corresponding magnetic interactions. The present approach allows for an approximate but very compact and yet accurate estimate of the local fields in individual particles situated in an arbitrary mesoscopic portion of the sample. In this way, we believe that our results represent a substantial progress and will help to develop more refined hybrid models to bridge the gap among the several length scales necessary to characterize MAE samples comprehensively. Currently, our approach is based on the dipole model to account for magnetic interactions. This adaptation represents a commonly applied modeling strategy towards MAEs. The here presented scheme, where different effects on different length scales are considered as nested into each other like in the famous Matryoshka toy, can be extendable in future works to account for a more adequate description of magnetic interactions among closely- located particles where the dipole approach reaches its limits [63]. As a prospective extension of the Matryoshka toy, upon introducing a fourth element which accounts for dipole corrections, it should be additionally helpful that such modifications are bound to very short ranges.

Beyond the computation benefit for application in simulations and/or hybrid models we want to emphasize that the cascading mean-field approach also provides a systematic framework to understand and to study the interplay between different effects directly. This is possible as our scheme provides a compact analytic, or at least semi-analytic, notation describing the leading effects on different length scales. Note, such analysis is achieved through the understanding that modifications in magnetization fields due to particle (re)arrangements, sample deformations, or altered external fields induce changes in the magnetic energy. Correspondingly, investigating the energetic minimum provides systematic insights to the behavior of an MAE. In a previous work [36] we used the leading order approximation of our approach for an efficient combination of theory and experiment to clarify some field-induced effects in MAEs. Furthermore, our scheme allows to study in detail the role of potential particle rearrangements and macroscopic deformations, as well as to consider subsequently the influence of different mechanical couplings and analyze the consequences. Accordingly, the current Cascading Mean-Field approach represents a consequent continuation of our previous work [32,33,34,52] towards a fundamental generalization of the idea to establish a unified theoretical description of the behavior of MAEs under an applied magnetic field.

## Figures and Tables

**Table 1:** Comparison between exact calculation and approximate results for the 100-particle configuration inside cubic boundaries, see top image in Figure 4, upon applying two different external fields $H_{0}$. The corresponding field components (x,y,z) are grouped in vectorial notation. The field quantities $H_{0}$, M and $M^{\left(1\right)}$ are denoted in A/m.

$H_{0}$	*M*	$M^{\left(1\right)}$	$\langle \delta M_{i}\rangle$	max$\left(\delta M_{i}\right)$	min$\left(\delta M_{i}\right)$
$\left(\begin{array}{c} 300,000\\ 0\\ 0 \end{array}\right)$	$\left(\begin{array}{c} 545,418\\ -2822\\ -2054 \end{array}\right)$	$\left(\begin{array}{c} 543,159\\ -2598\\ -1780 \end{array}\right)$	$9.67\times 10^{-3}$	$2.16\times 10^{-2}$	$2.0\times 10^{-3}$
				
$\left(\begin{array}{c} 300,000\\ 300,000\\ 100,000 \end{array}\right)$	$\left(\begin{array}{c} 466,232\\ 464,915\\ 154,510 \end{array}\right)$	$\left(\begin{array}{c} 466,187\\ 465,122\\ 154,893 \end{array}\right)$	$4.92\times 10^{-3}$	$9.85\times 10^{-3}$	$8.48\times 10^{-4}$

**Table 2:** Comparison between exact calculation and approximate results for the 100-particle configuration inside elongated cylindrical boundaries, see bottom image in Figure 4, upon applying the same two external fields $H_{0}$ as in the cubic case. The corresponding field components (x,y,z) are grouped in vectorial notation. Magnetic and magnetization fields are denoted in A/m.

$H_{0}$	*M*	$M^{\left(1\right)}$	$\langle \delta M_{i}\rangle$	max$\left(\delta M_{i}\right)$	min$\left(\delta M_{i}\right)$
$\left(\begin{array}{c} 300,000\\ 0\\ 0 \end{array}\right)$	$\left(\begin{array}{c} 585,871\\ 1780\\ -3357 \end{array}\right)$	$\left(\begin{array}{c} 584,846\\ 1556\\ -3579 \end{array}\right)$	$6.44\times 10^{-3}$	$1.44\times 10^{-2}$	$6.93\times 10^{-4}$
				
$\left(\begin{array}{c} 300,000\\ 300,000\\ 100,000 \end{array}\right)$	$\left(\begin{array}{c} 505,700\\ 440,194\\ 141,853 \end{array}\right)$	$\left(\begin{array}{c} 505,471\\ 440,561\\ 141,820 \end{array}\right)$	$4.45\times 10^{-3}$	$1.28\times 10^{-2}$	$7.98\times 10^{-4}$

**Figure A1:**
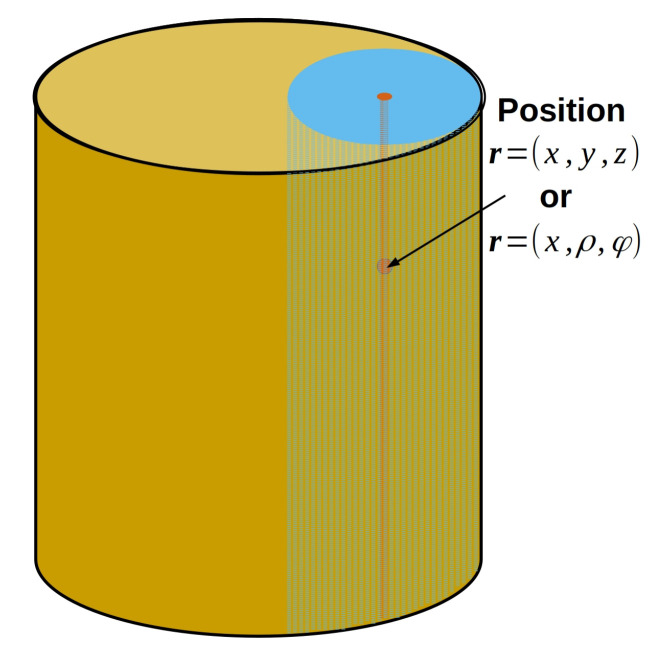
Sketch of the decomposition of a cylindrical sample to solve the integral in Equation (69).

## Data Availability

On inquiry, the data presented in this study is available from the authors.

## Supplementary Materials

The following are available online at https://www.mdpi.com/article/10.3390/polym13091372/s1.

## Appendix A

The calculation of the 3-dimensional integral in Equation (69) is not straightforward, especially due the necessity of cutting off the pole at $r=r^{\prime }$. In the following we derive partially analytic closed forms, i.e., we may leave some parts of Equation (69) as numerically derived integrations. There may be yet several more mathematically involved solution methods allowing for representations with more or full analytically closed terms. However for the present example we consider partially numerical forms as sufficient.

In Figure A1 we sketch our decomposition scheme of the integral Equation (69) into three parts.

The first part refers to the innermost sub-cylinder, red colored in Figure A1, and accounts for the contribution right above, resp. below, the position r. The second part, blue colored in Figure A1, captures the encapsulating cylinder around the red one where yet a full angular integration, i.e., $\varphi^{\prime }\in \left[0,2\pi \right]$ is viable. Finally, the third part describes the non-shaded rest in Figure A1. With such decomposition we find for $G_{xx}$:

(A1) $$\begin{array}{c} G_{xx}=-\frac{2}{3}+\frac{\frac{\Gamma }{2}\left(\frac{1}{2}+X\right)}{\sqrt{{\left(\frac{1}{2}-P\right)}^{2}+\Gamma^{2}{\left(\frac{1}{2}+X\right)}^{2}}}+\frac{\frac{\Gamma }{2}\left(\frac{1}{2}-X\right)}{\sqrt{{\left(\frac{1}{2}-P\right)}^{2}+\Gamma^{2}{\left(\frac{1}{2}-X\right)}^{2}}}\\ -F\left(\Gamma (1-2X),P\right)-F\left(\Gamma (1+2X),P\right)\;. \end{array}$$

Here, we use dimensionless cylindrical coordinates $X=\frac{x}{L}$ and $P=\frac{\rho }{D}$ with *L* the length, or height, of the cylinder and *D* its diameter. The aspect ratio of the cylinder is then $\Gamma =\frac{L}{D}$. In the first line in Equation (A1) the analytically closed form for the first and the second part of the decomposition (red- and blue-colored sub-cylinder in Figure A1) are given. In the second line the remaining part is condensed in function F(W,P) which reads here:

(A2) $$F\left(W,P\right)=\frac{W}{2\pi }\int_{1-2P}^{1+2P}d\eta \;\eta \frac{\arccos\left(\frac{4P^{2}+\eta^{2}-1}{4P\eta }\right)}{{\left(\eta^{2}+W^{2}\right)}^{3/2}}\;.$$

This 1-dimensional integral can be solved numerically.

For the cross-term $G_{x\rho }$, we find the following form:

(A3) $$\begin{array}{cc} G_{x\rho }= & \frac{A\left(\frac{4P}{1+4\Gamma^{2}{\left(\frac{1}{2}+X\right)}^{2}+4P^{2}}\right)}{2\pi \sqrt{1+4\Gamma^{2}{\left(\frac{1}{2}+X\right)}^{2}+4P^{2}}}-\frac{A\left(\frac{-4P}{1+4\Gamma^{2}{\left(\frac{1}{2}+X\right)}^{2}+4P^{2}}\right)}{2\pi \sqrt{1+4\Gamma^{2}{\left(\frac{1}{2}+X\right)}^{2}+4P^{2}}}\\ + & \frac{A\left(\frac{-4P}{1+4\Gamma^{2}{\left(\frac{1}{2}-X\right)}^{2}+4P^{2}}\right)}{2\pi \sqrt{1+4\Gamma^{2}{\left(\frac{1}{2}-X\right)}^{2}+4P^{2}}}-\frac{A\left(\frac{4P}{1+4\Gamma^{2}{\left(\frac{1}{2}-X\right)}^{2}+4P^{2}}\right)}{2\pi \sqrt{1+4\Gamma^{2}{\left(\frac{1}{2}-X\right)}^{2}+4P^{2}}}\;. \end{array}$$

The function A(V) is defined as:

(A4) $$A\left(V\right)={}_{3}F_{2}\left(\frac{1}{4},\frac{3}{4},1;\frac{1}{2},\frac{3}{2};V^{2}\right)+\frac{\left(1+V\right)E\left(\frac{2V}{1+V}\right)-K\left(\frac{2V}{1+V}\right)}{V\sqrt{1+V}}\;,$$

where ${}_{3}F_{2}$ denotes the generalized hypergeometric function, *E* represents the complete elliptic integral of the second kind, and *K* represents the complete elliptic integral of the first kind.
